# Salient features of the *aza*-Wacker cyclization reaction

**DOI:** 10.1039/d0sc02554b

**Published:** 2020-07-21

**Authors:** Annu Anna Thomas, Someshwar Nagamalla, Shyam Sathyamoorthi

**Affiliations:** Department of Medicinal Chemistry, University of Kansas Lawrence KS USA ssathyam@ku.edu

## Abstract

The intramolecular *aza*-Wacker reaction has unparalleled potential for the site-selective amination of olefins, but it is perhaps underappreciated relative to other alkene oxidations. The first part of this review makes the distinction between classical and tethered *aza*-Wacker cyclization reactions and summarizes examples of the latter. The second portion focuses on developments in asymmetric *aza*-Wacker cyclization technology. The final part of the review summarizes applications of all classes of *aza*-Wacker cyclization reactions to natural product assembly.

## Abstract 2

The *aza*-Wacker cyclization reaction is a powerful strategy for alkene amination.

## Graphical abstract

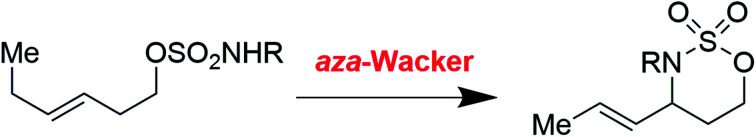

## 1. Introduction

The oxidative functionalization of alkenes remains a central area of focus within the synthetic community.^1–3^ Within this extensive field, Wacker oxidations of alkenes into ketones^4^ and Wacker-type cyclizations of alkenyl alcohols into furans have been extensively investigated. In sharp contrast, the corresponding oxidative cyclization of alkenyl amines, generally termed *aza*-Wacker reactions, has received less attention.^5–7^ This is unfortunate, as intramolecular *aza*-Wacker reactions offer unparalleled opportunity for the site-selective amination of alkenyl moieties.

**Fig. 1:**
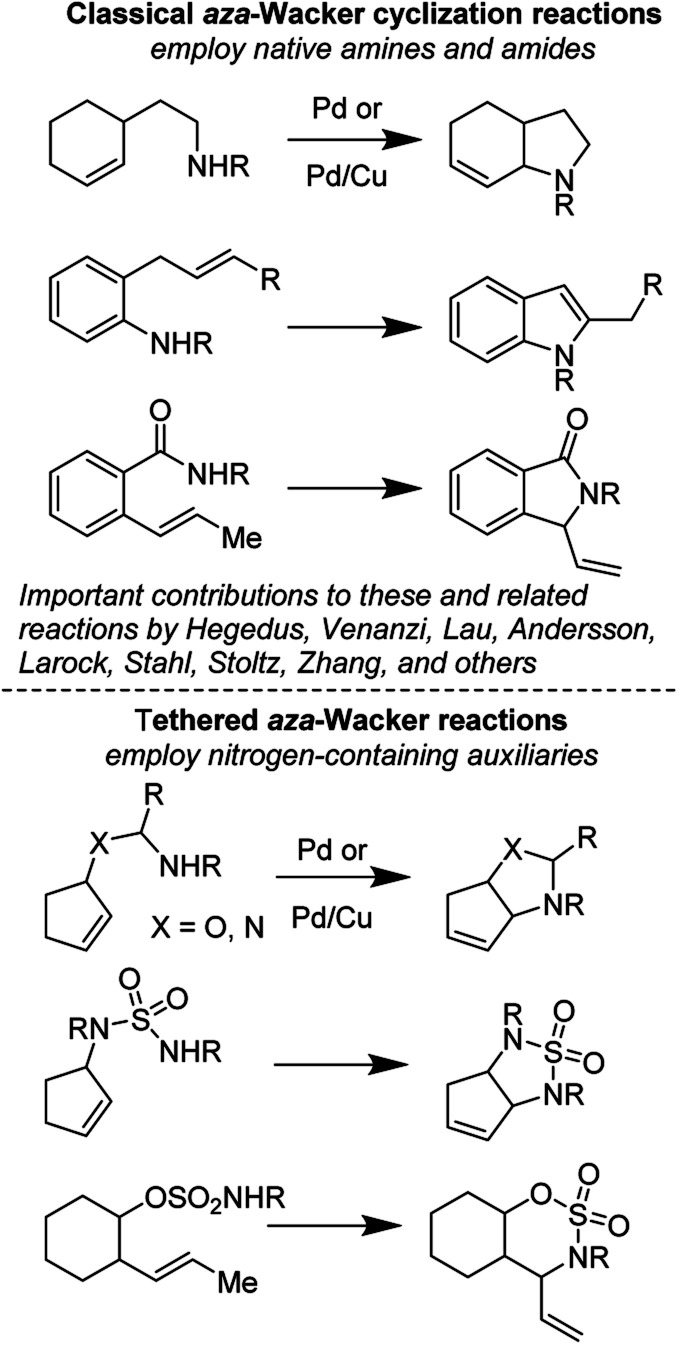
Classical and tethered *aza*-Wacker cyclization reactions.

Aspects of *aza*-Wacker chemistry have been summarized in other elegant reviews, often within the context of larger accounts of palladium-catalyzed reactions.^1,3,5^ The purpose of this review is to highlight past progress and future opportunities within the field of *aza*-Wacker cyclization reactions. Specifically, we will focus on three categories of the intramolecular *aza*-Wacker reaction:

(1) *Tethered aza-Wacker cyclizations*: it is generally believed that intramolecular *aza*-Wacker cyclizations generally proceed by Pd(ii)-assisted attack of an amine onto an alkene moiety followed by β-hydride abstraction, reductive elimination, and re-oxidation of Pd(0) to Pd(ii). It is important to note however that Pd(ii) assisted C–H cleavage forming a π-allyl Pd complex followed by attack of an amine nucleophile often furnishes identical products; distinguishing between these two competing mechanisms has been the foundation of many elegant physical organometallic experiments. Within the *aza*-Wacker reaction class, there are two fundamental subsets: (A) *Classical aza-Wacker chemistry*, which utilizes native amines and amides for the oxidative cyclization event and (B) *Tethered aza-Wacker chemistry*, which makes use of nitrogen-containing auxiliaries that can be easily appended to other functional groups in the molecule followed by oxidative cyclization and then removal. This distinction has not previously been made in the literature, but we find this essential in order to organize future efforts in this exciting field (Fig. 1). The importance of a tethered reaction is that it frees the synthetic practitioner from the constraint of needing a pre-existing C–N bond in order to forge a new one. This greatly expands the contexts in which the *aza*-Wacker cyclization can be employed.

(2) *Enantioselective aza-Wacker reactions*: the development of enantioselective *aza*-Wacker chemistry is an exciting, burgeoning field. Currently, few examples of this challenging transformation exist. The reasons for this are numerous and stem from the inherent reversibility of amino-palladation, competing *cis*/*trans* nucleopalladation, and a general lack of asymmetric ligands for Pd(ii)–Pd(0) redox manifolds. We will describe existing enantioselective *aza*-Wacker processes.

(3) *aza-Wacker chemistry in natural product synthesis*: there are currently only a handful of examples of *aza*-Wacker chemistry utilized in the assembly of natural products. This is in sharp contrast to other alkene and alkane oxidation reactions, especially C–H amination technology.^8^ We will summarize existing examples, which we believe illustrate the great precision with which *aza*-Wacker chemistry allows for the installation of nitrogen functionality. The wider adoption of *aza*-Wacker reactions, especially tethered *aza*-Wacker cyclizations, by the synthetic community would represent an important shift in the logic of nitrogen insertion during complex molecule assembly.

We hope that calling attention to these facets of the *aza*-Wacker cyclization reaction will inspire creative developments in synthetic methodology and elegant applications in total synthesis.

## 2. Tethered *aza*-Wacker cyclization reactions

### 2.1 Aminal tethered *aza*-Wacker cyclization (1994)

**Fig. 2:**
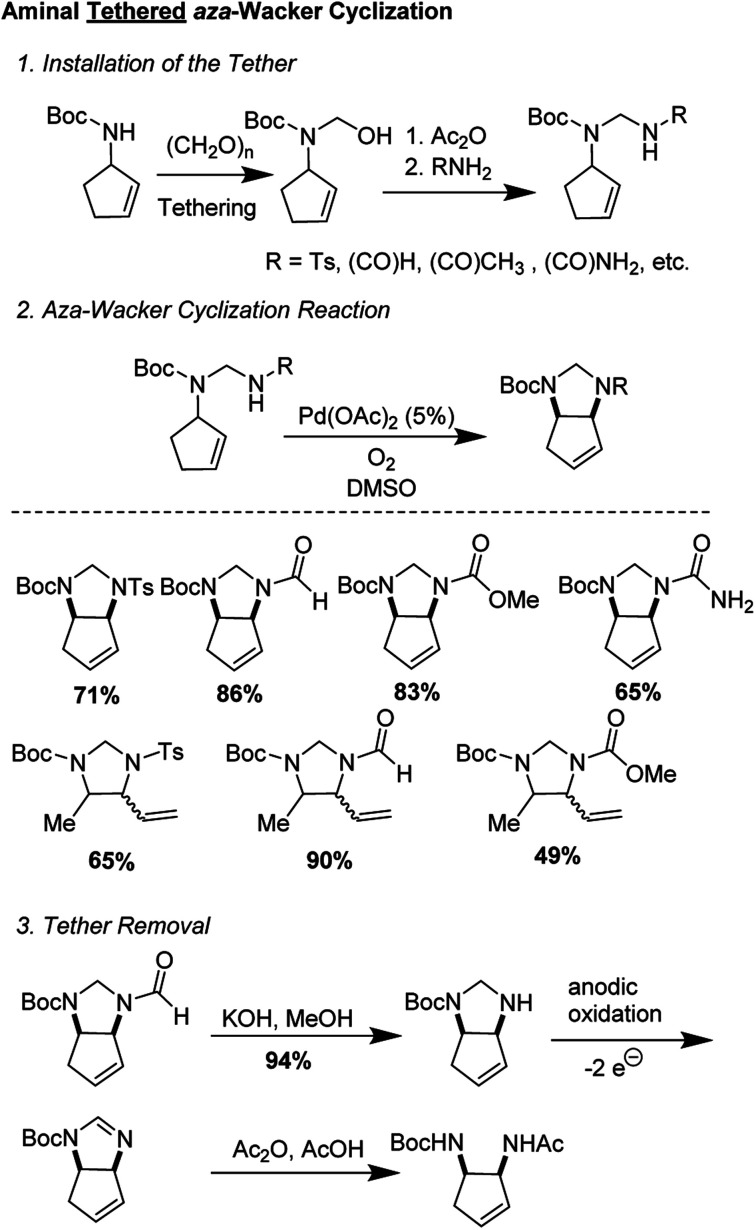
Hiemstra's aminal tethered *aza*-Wacker cyclization.

One of the first examples of a tethered *aza*-Wacker cyclization reaction was disclosed by Hiemstra and co-workers in 1994,^9^ who showed that an aminal tether was competent for the formation of vicinal diamines (Fig. 2). The tether was appended in three steps from an allylic monoamine, involving synthesis of an *N*,*O*-acetal followed by nucleophilic displacement of the O-group by a nitrogen containing functional handle, such formamide, acetamide, methyl carbamate, and *p*-toluenesulfonamide. Aminals were prepared from either *N*-Boc-2-cyclopentenyl amine and *N*-Boc-3-pent-2-(*E*)-enylamine. *aza*-Wacker cyclization then proceeded upon treatment with 5 mol% Pd(OAc)_2_ in DMSO under 1 atm O_2_. Of the various nucleophiles tested, it was found that aminals derived from formamide were the most competent. Tether removal proved to be surprisingly difficult and required KOH hydrolytic removal of the formamide group, electrocatalytic conversion into the amidine, and transformation into the protected diamine by reaction with acetic anhydride in 1 : 1 acetic acid/water.

### 2.2 Amide tethered *aza*-Wacker cyclization (2004)

**Fig. 3:**
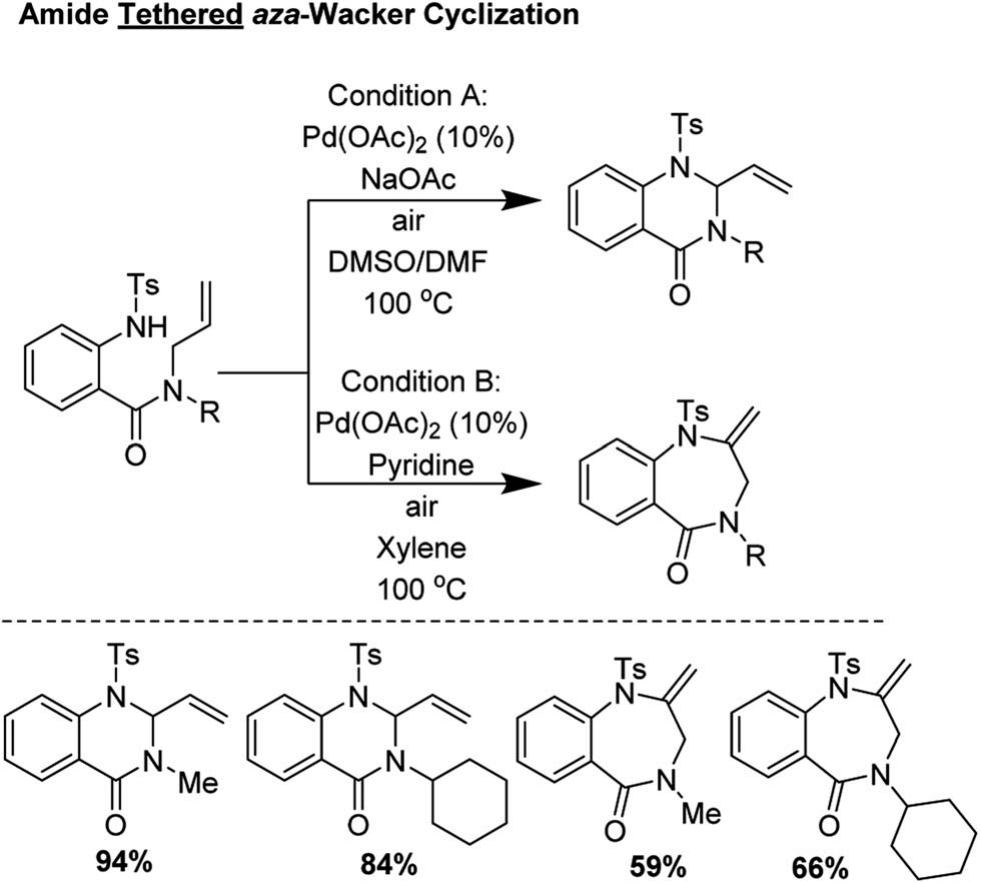
Divergent reaction conditions for the formation of quinazolin-4-ones and 1,4-benzodiazepin-5-ones.

Broggini and coworkers reported an amide tethered *aza*-Wacker cyclization for the synthesis of 1,4-benzodiazepin-5-ones from tosylated *N*-allyl-anthranilamides (Fig. 3); importantly, they found that conditions could be tuned to also furnish quinazolin-4-one products.^10^ They found that choice of solvent had a critical effect in biasing the formation of one of these heterocycles relative to the other. In the presence of 10 mol% Pd(OAc)_2_, with a polar, aprotic solvent like DMSO or DMF, quinazolin-4-ones were formed in good yield; conversely, when xylene/pyridine was used, 1,4-benzodiazepin-5-ones were furnished preferentially. The authors found that the presence of base, exposure to air, and choice of protecting group on the amine were essential for product formation.

**Fig. 4:**
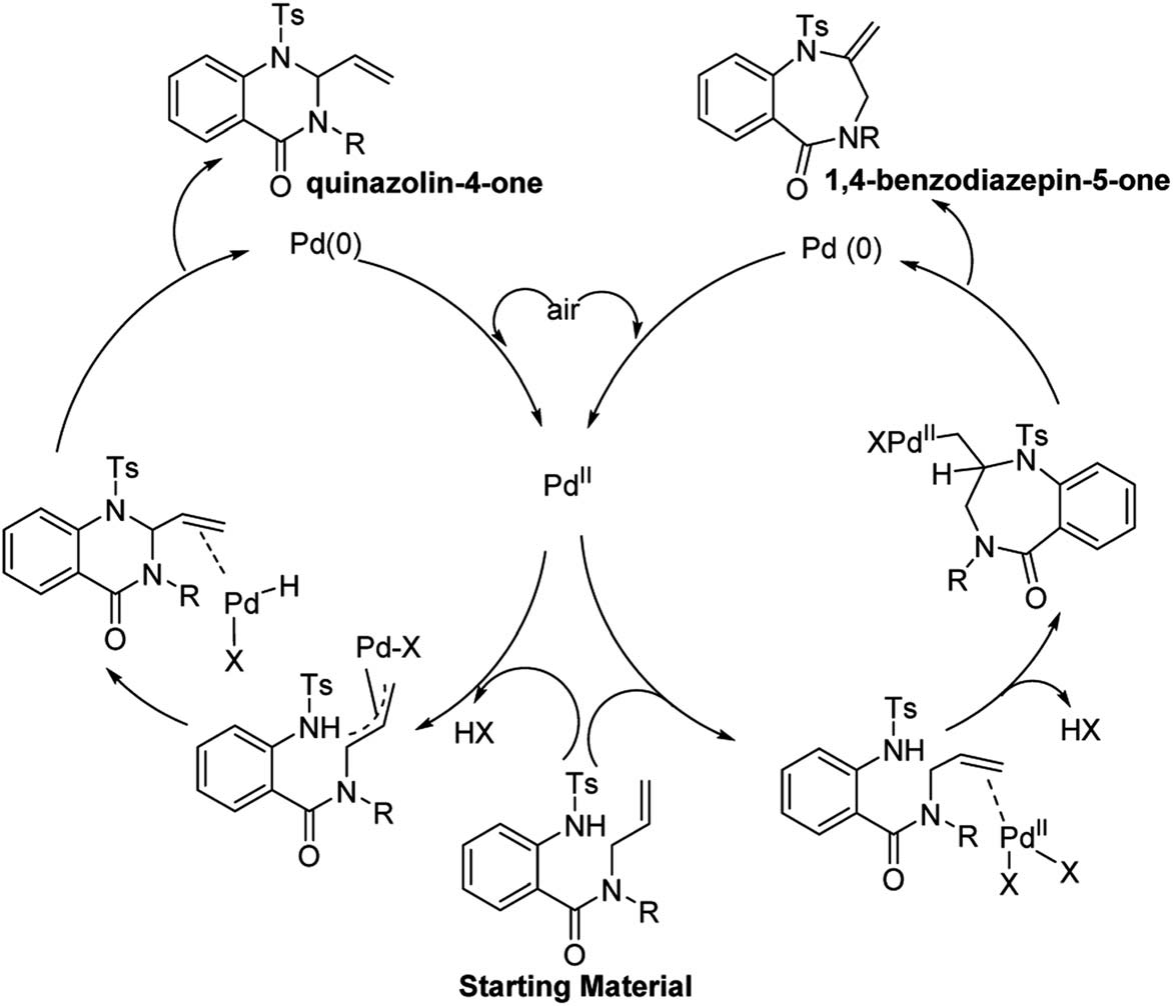
Proposed reaction mechanism.

**Fig. 5:**
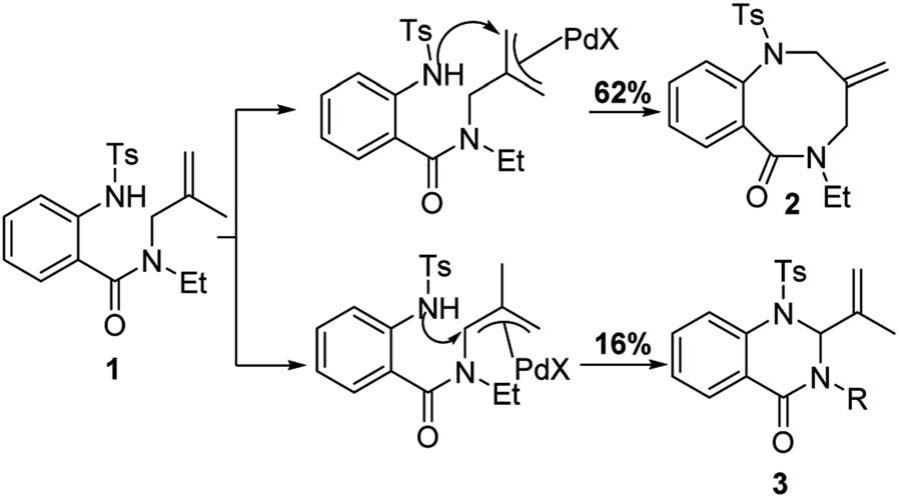
Mechanistic studies using probe substrate **1**.

Under standard conditions, for the formation of 6-membered quinazolin-4-one products, the authors hypothesized that the first step in the reaction pathway is palladium-mediated allylic C–H cleavage to form an η-3-allyl-palladium complex (Fig. 4). Subsequent nucleophilic attack by the pendant protected amine would furnish the 6-membered heterocyclic ring. This hypothesis was further bolstered by using probe substrate **1** (Fig. 5). Here, synthesis of products **2** and **3** would *necessitate* prior formation of an η-3-allyl-palladium complex. For the formation of 7-membered 1,4-benzodiazepin-5-one products, the authors proposed that the first step in the reaction pathway was a nucleopalladation reaction followed by β-hydride elimination and reductive elimination (Fig. 4).

### 2.3 Sulfamide tethered *aza*-Wacker cyclization (2010)

**Fig. 6:**
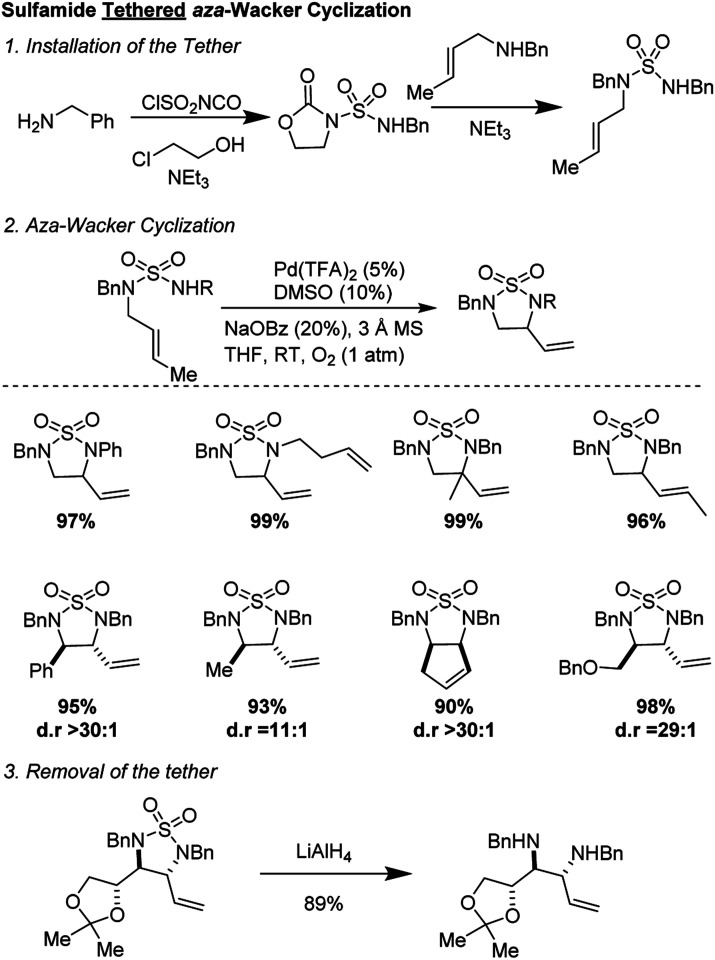
Stahl's sulfamide tethered *aza*-Wacker cyclization.

In 2010, Stahl and co-workers reported an elegant synthesis of 1,2-diamines using a sulfamide tether (Fig. 6).^11^ Starting materials were synthesized in a two-step sequence from mono-amine precursors. Oxidative cyclization occurred in good yields and with excellent diastereoselectivity (often >30 : 1) using the reagent combination of 5 mol% Pd(TFA)_2_, 10 mol% DMSO, 20 mol% NaOBz in THF under 1 atmosphere of O_2_. The reaction was tolerant of a range of substituents on the nucleophilic nitrogen and worked on diverse alkenyl amines, both open-chain and cyclic. The sulfonyl group could be removed by treatment with LiAlH_4_ to furnish highly valuable diamine products.

**Fig. 7:**
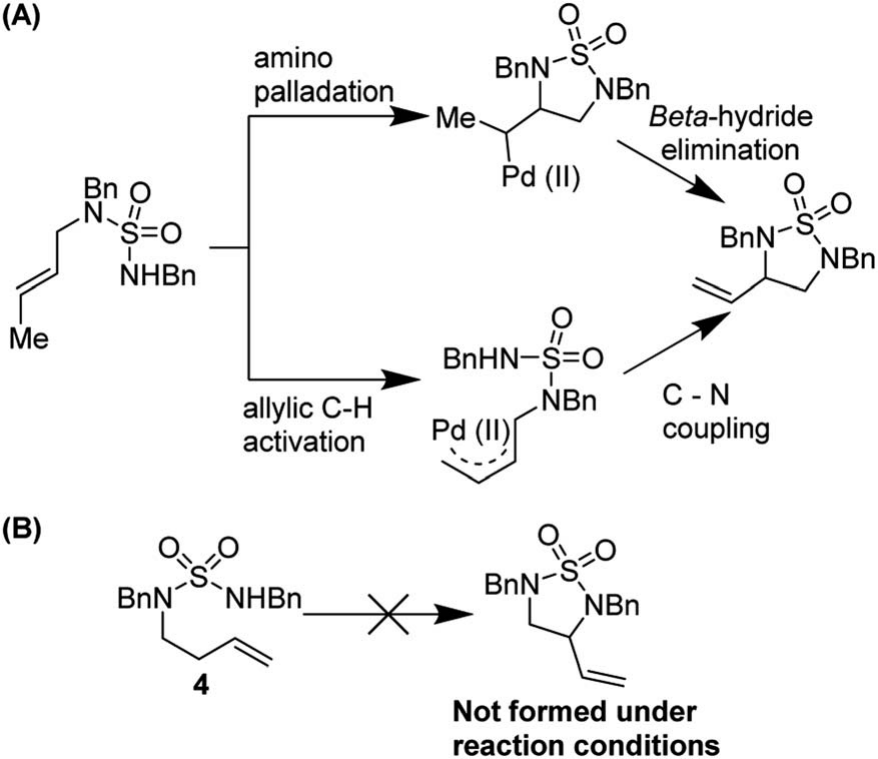
(A) Two possible reaction pathways (B) substrate probe experiment shows that nucleopalladation is more likely.

Stahl and co-workers studied the mechanism of this reaction thoroughly (Fig. 7). Using variable temperature ^1^H NMR studies, they showed that DMSO can coordinate to Pd(ii) *via* both sulfur and oxygen and is kinetically labile. They noted that two reaction pathways are possible but were able to rule out an allylic C–H activation mechanism by employing probe substrate **4**.

### 2.4 Carbamate tethered *aza*-Wacker cyclization (2012)

**Fig. 8:**
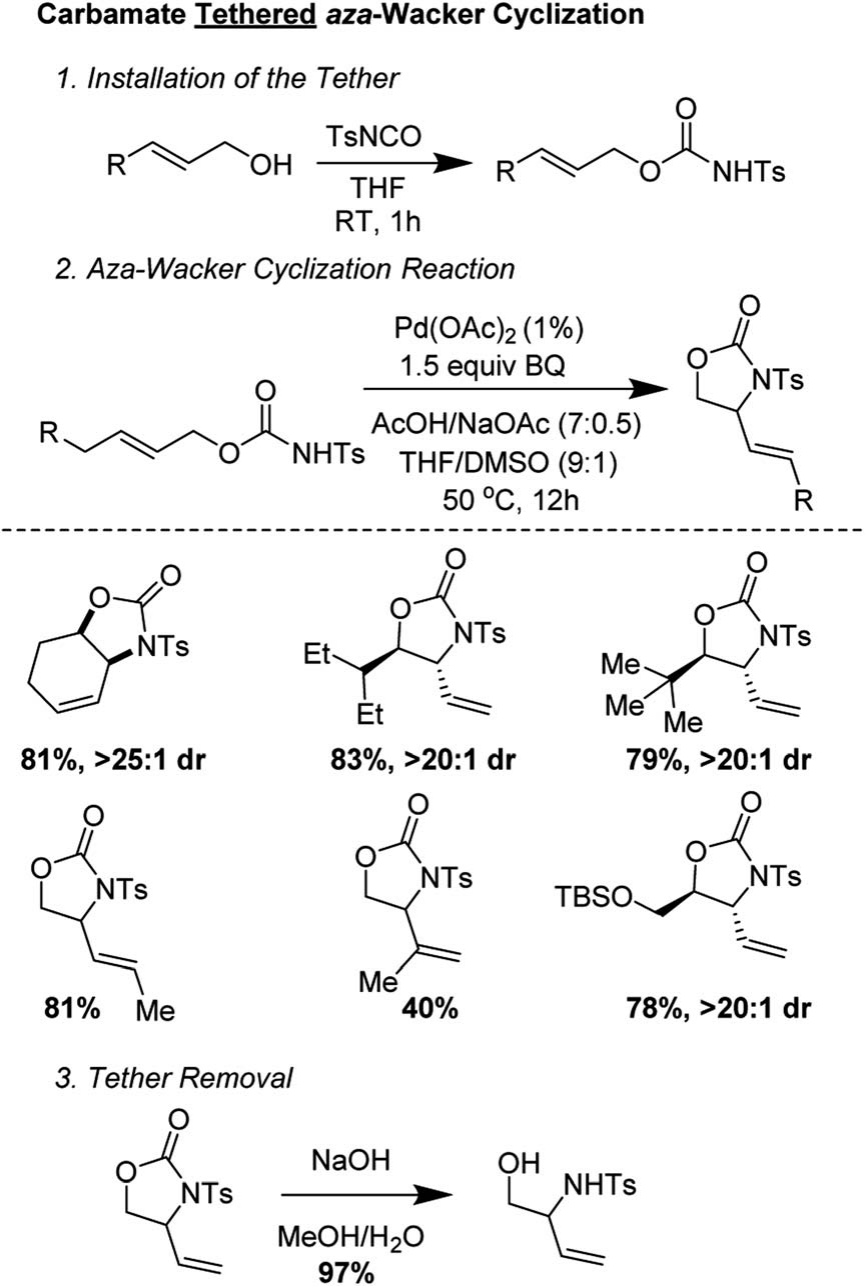
Backvall's carbamate tethered *aza*-wacker cyclization.

Bäckvall and co-workers showed that carbamate tethers were competent for *aza*-Wacker cyclization reactions (Fig. 8).^12^ Despite using very low palladium loadings (1 mol%), the reactions proceeded in good yield and with excellent diastereoselectivity. Interestingly, they found that the starting olefin geometry had a profound effect on reaction progress; carbamates derived from (*Z*)-allylic alcohols cyclized much more reliably than those from the corresponding (*E*) isomer. The reaction worked best for carbamates derived from disubstituted allylic alcohols; reaction times were markedly extended for those derived from trisubstituted allylic alcohols.

**Fig. 9:**
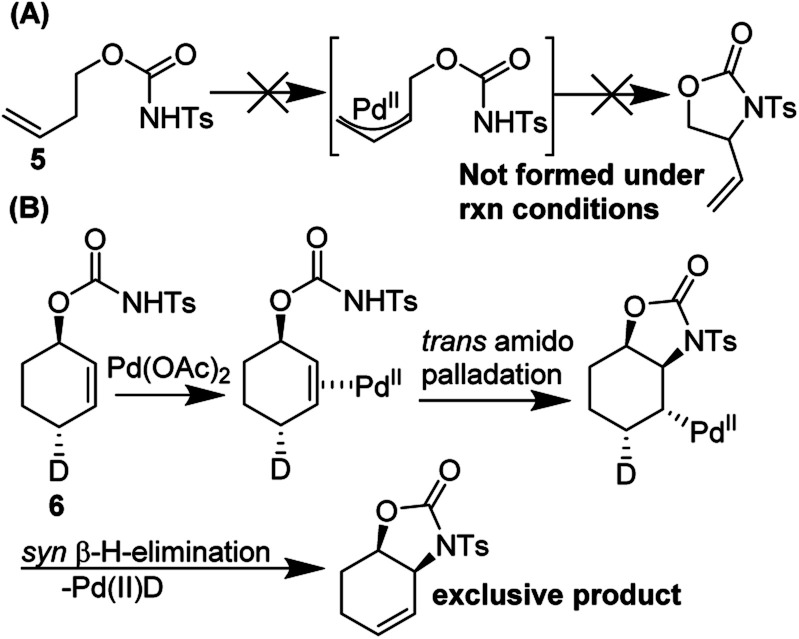
Backvall's experiments suggest (A) a nucleopalladation mechanism and (B) *trans*-amidopalladation.

They conducted an elegant series of experiments in order to elucidate the reaction pathway (Fig. 9). As no reaction was observed with probe substrate **5**, they concluded that amido-palladation was more likely as a first step compared to allylic C–H cleavage to form a π-allyl complex. Further, using deuterated substrate **6**, they were able to determine that the reaction likely proceeds *via trans*-amidopalladation followed by *syn*-β-hydrogen-elimination.

### 2.5 *N*,*O*-Acetal tethered *aza*-Wacker cyclization (2013)

**Fig. 10:**
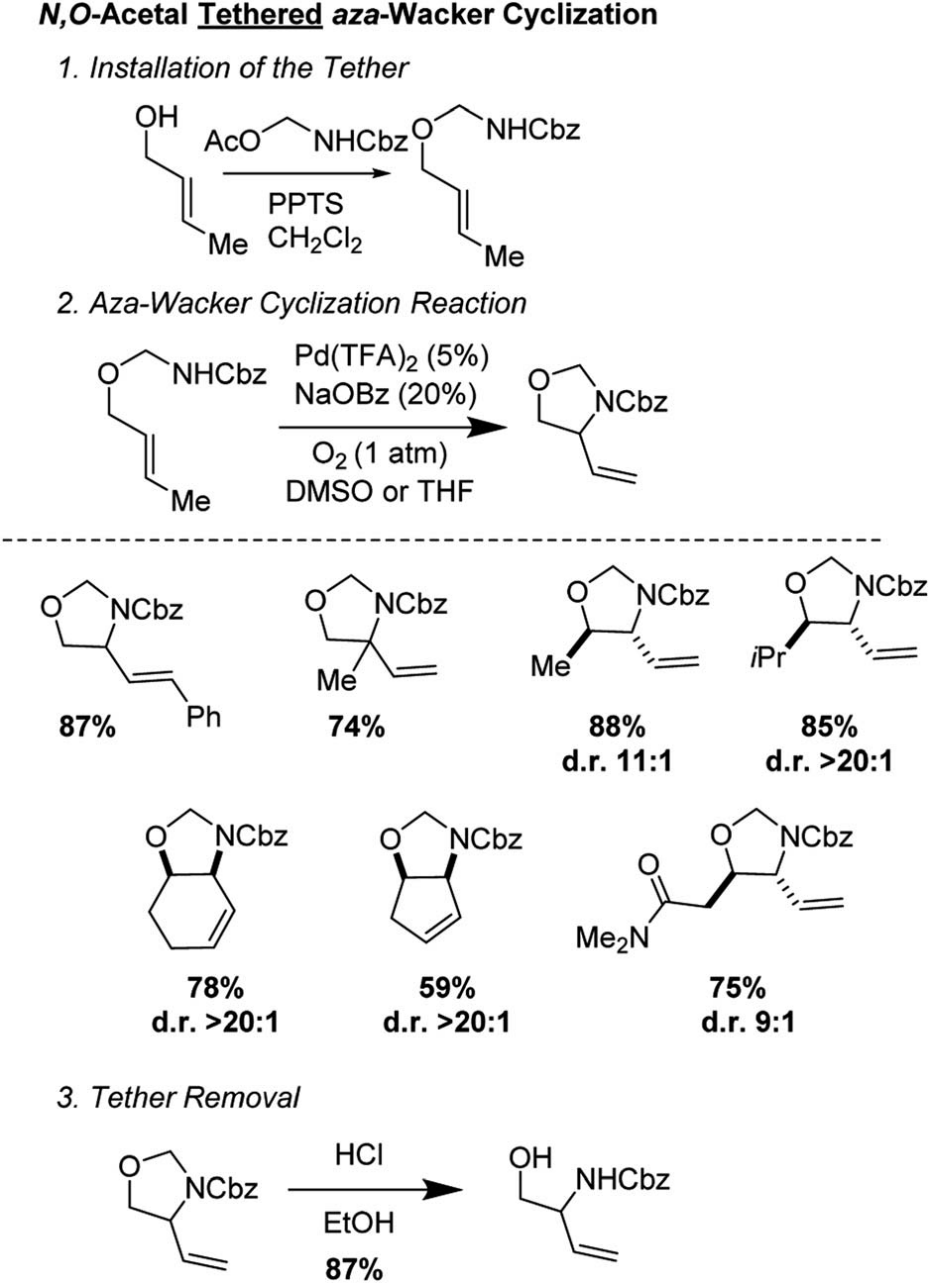
Stahl's *N*,*O*-acetal tethered *aza*-Wacker cyclization.

**Fig. 11:**
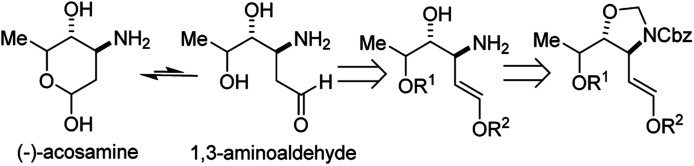
Redox-relay approach to (−)-acosamine.

Stahl and co-workers developed an elegant approach for the synthesis of masked 1,2-aminoalcohols using an *N*,*O*-acetal tethered *aza*-Wacker cyclization reaction (Fig. 10).^13^ The cyclization reaction was highly selective for the formation of 5-membered rings and exhibited excellent diastereoselectivity. The tether was appended in a single step reaction by reacting various allylic alcohols with Cbz-amino-methyl acetate and could be facilely removed using HCl in MeOH. They were able to apply this reaction successfully in a redox-relay synthesis (Fig. 11) of the 2-deoxy-3-aminosugar-(−)-acosamine (see Section 4 of this review for full details of the synthesis).

### 2.6 *N*-Sulfonyl-amide tethered *aza*-Wacker cyclization (2017)

**Fig. 12:**
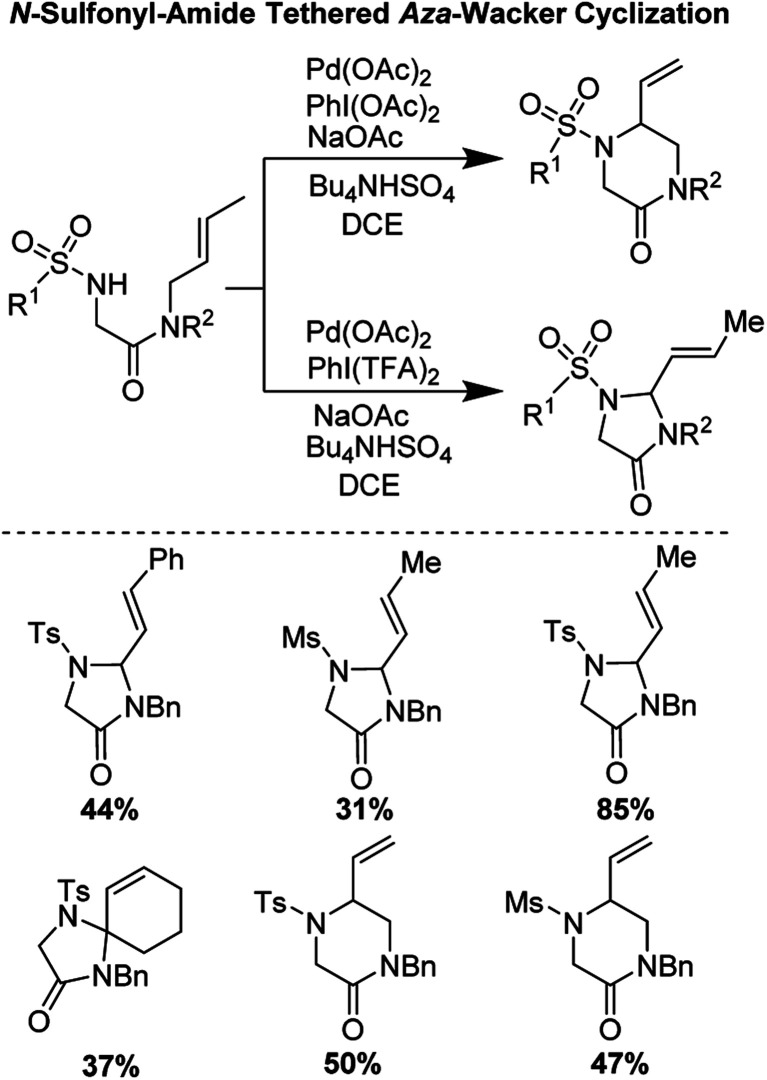
Poli and Broggini's *N*-sulfonyl-amide tethered *aza*-Wacker cyclization.

In 2017, Poli, Broggini, and co-workers reported an interesting *N*-sulfonyl-amide tethered *aza*-Wacker cyclization reaction for the synthesis of imidazolidine-4-one and piperazin-2-one heterocycles (Fig. 12).^14^ They found that by tuning the reaction conditions, namely the nature of the hypervalent iodine oxidant used, they could bias the formation of one heterocyclic product relative to the other.

**Fig. 13:**
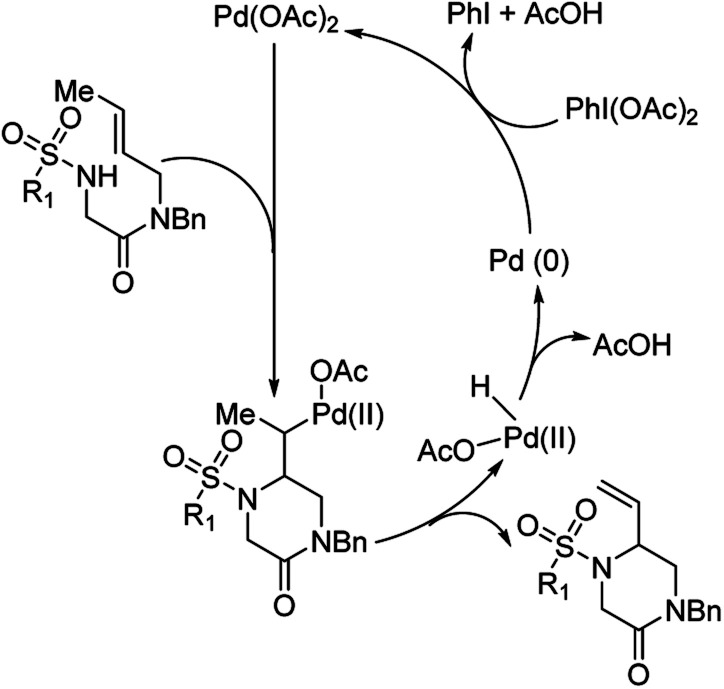
Proposed catalytic cycle for the formation of piperazinone products.

They hypothesized that for the formation of 6-membered piperazinone products, initial aminopalladation lead to the formation of an alkyl-Pd species which was followed by β-hydrogen abstraction and reductive elimination (Fig. 13). PhI(OAc)_2_ promoted oxidation of Pd(0) to Pd(ii). PhI(TFA)_2_, however, promoted allylic C–H bond cleavage allowing for a pathway favoring 5-membered imidazolidinone formation.

### 2.7 *N*-Ts hydrazine tethered enantioselective *aza*-Wacker cyclization (2018)

**Fig. 14:**
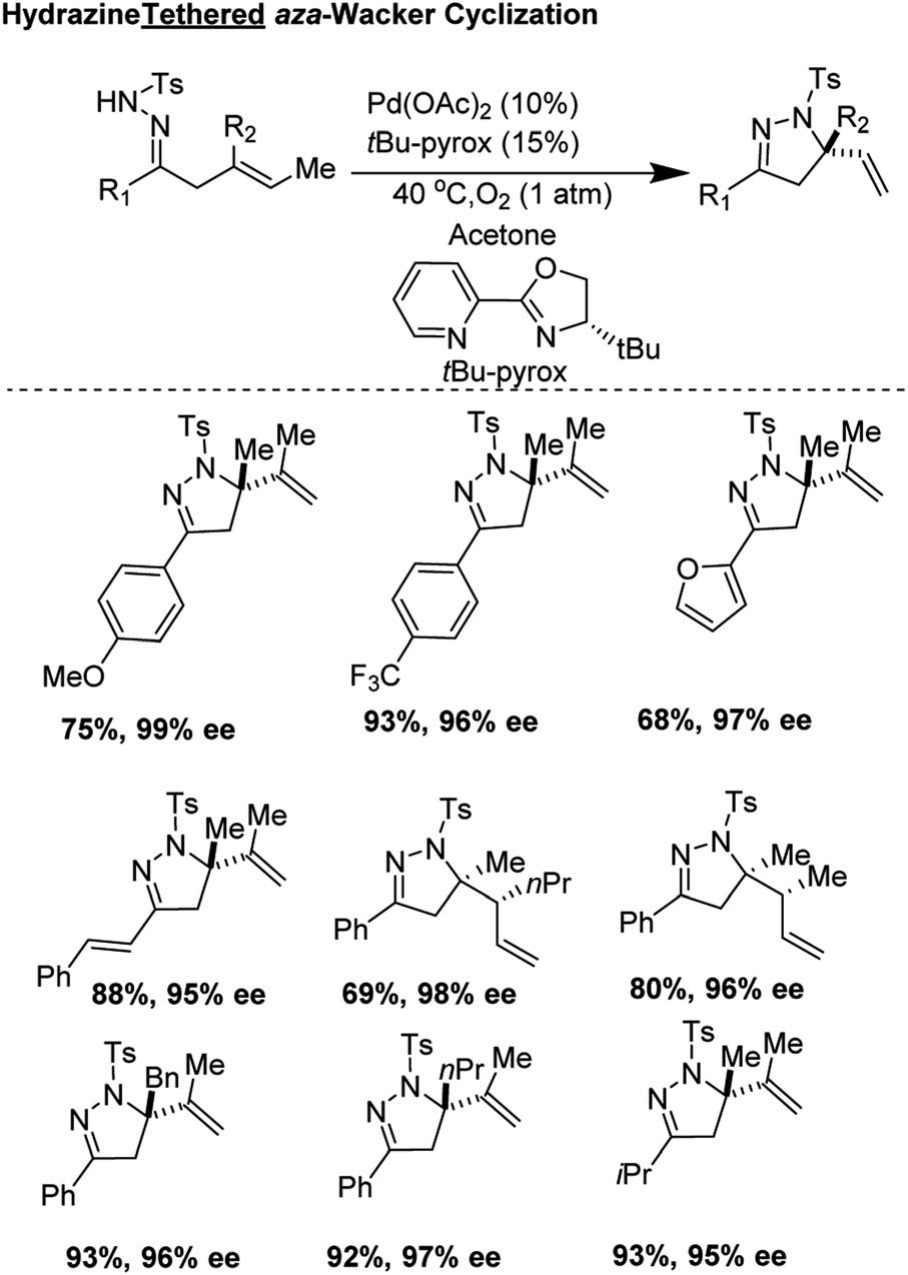
Zhang and Yang's hydrazine tethered asymmetric *aza*-Wacker cyclization.

In 2018, Yang, Zhang, and co-workers developed an elegant asymmetric *aza*-Wacker cyclization using *N*-tosyl hydrazine as a tether which could be attached to ketone moieties (Fig. 14).^15^ The reaction was tolerant of a variety of olefin substrates; the authors found that with the combination of Pd(OAc)_2_ and the chiral ligand *t*Bu-Pyrox, enantiomeric excesses as high as 98% could be obtained. In the majority of substrates, quaternary stereocenters could be formed in good yield and excellent enantioselectivity. In certain substrates, products with two vicinal stereocenters could be generated in one step as a single diastereomer, a highly challenging transformation within the realm of *aza*-Wacker chemistry.

**Fig. 15:**
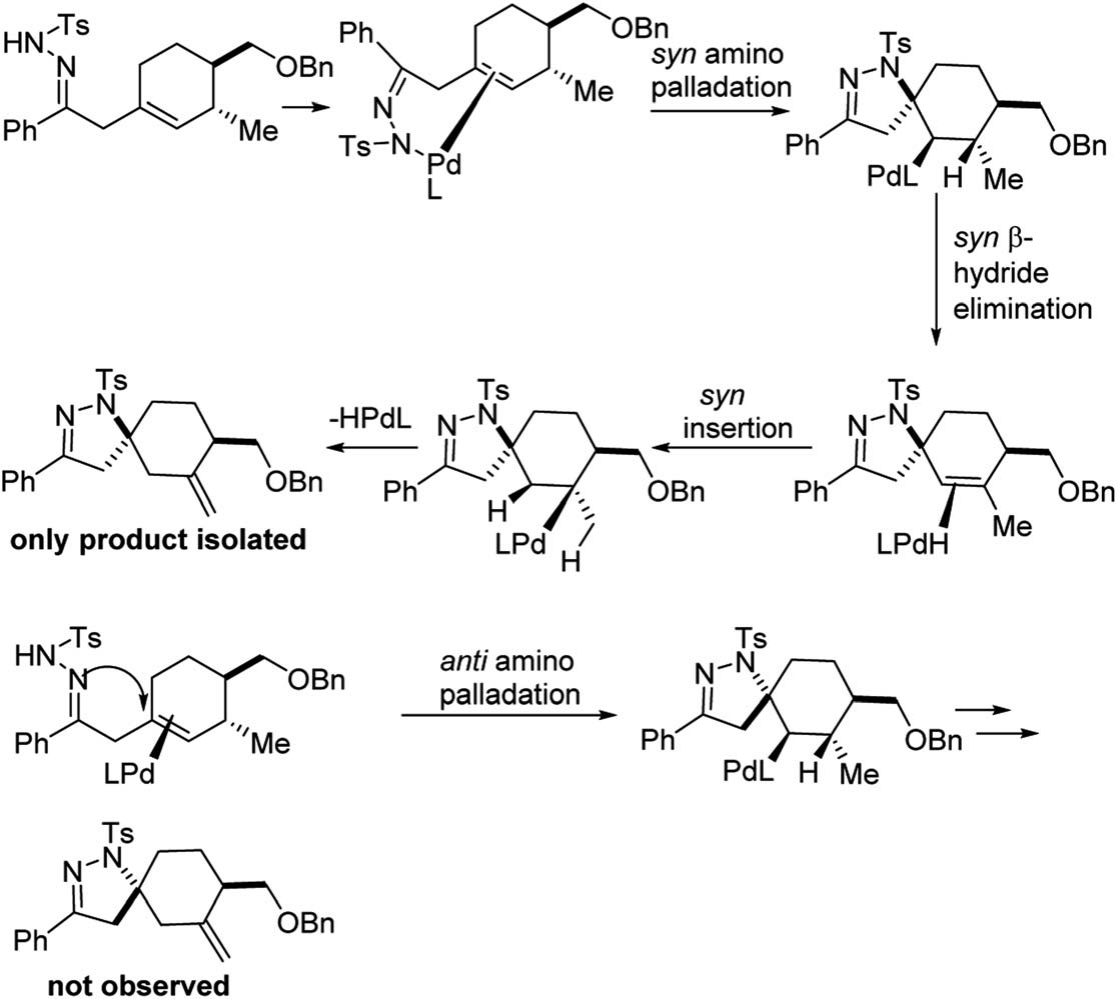
Mechanistic studies suggest a *syn*-amino-palladation pathway.

The authors carefully examined the mechanism of the reaction using a combination of substrate probe studies and isotope labelling experiments (Fig. 15). Based on this, they hypothesized that this *aza*-Wacker cyclization proceeds *via syn*-amino-palladation.

### 2.8 Sulfamate tethered *aza*-Wacker cyclization (2020)

**Fig. 16:**
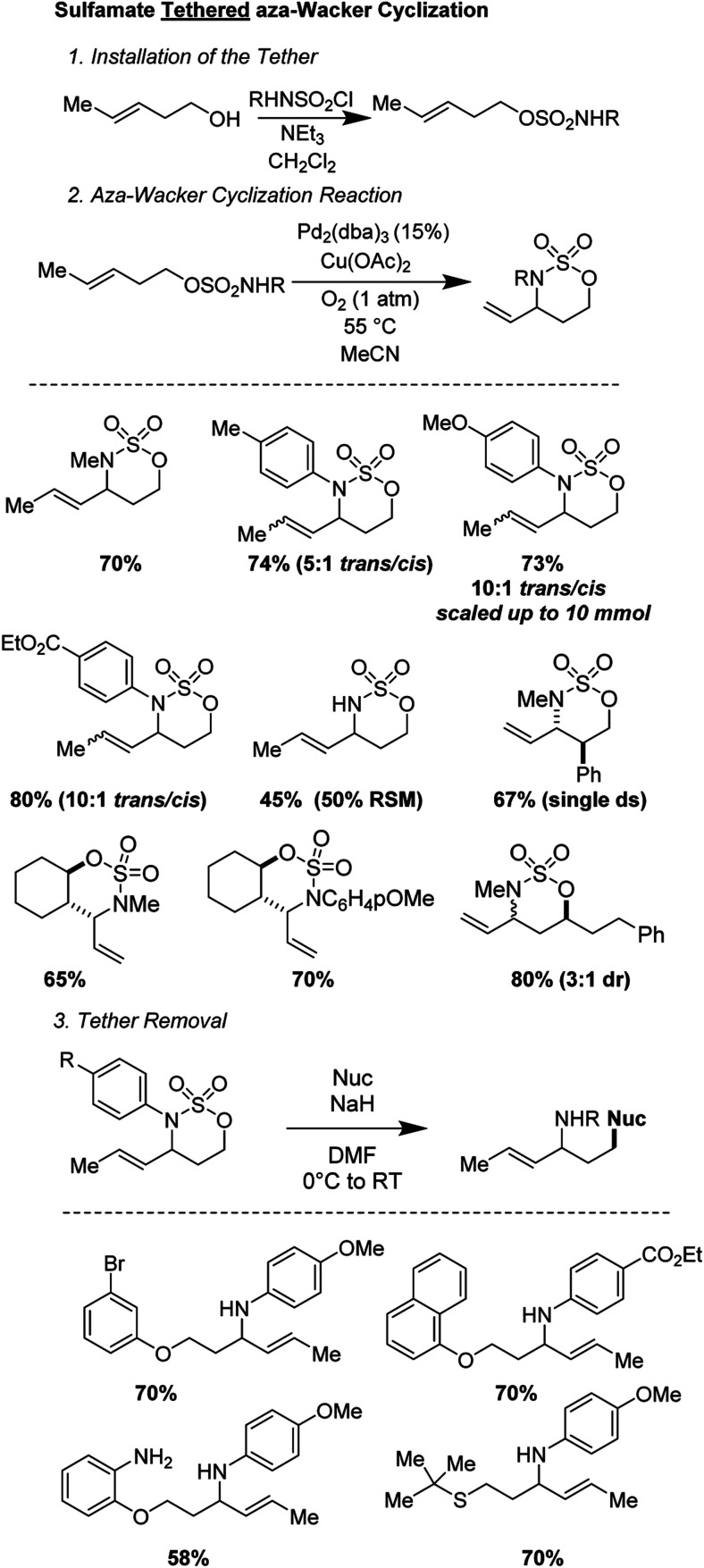
Sathyamoorthi's tethered *aza*-Wacker cyclization.

Sathyamoorthi and Shinde recently disclosed a sulfamate tethered *aza*-Wacker cyclization reaction that reliably forms six-membered oxathiazinane heterocyclic rings in good yields and with reasonable (3 : 1) to excellent (>20 : 1) diastereoselectivities (Fig. 16).^16^ The sulfamate auxiliary could be conveniently appended to a variety of alcohols and cyclized onto diverse pendant alkenes. The reaction utilizes the unusual reagent combination of Pd_2_(dba)_3_ along with Cu(OAc)_2_ under 1 atm of O_2_. Diverse aryl and alkyl groups attached to the nitrogen of the sulfamate were tolerated. In addition, even when the scale was increased from 0.2 mmol of substrate (∼50 mg) to 10 mmol (∼2.8 g), the yield did not suffer.^17^ The oxathiazinane ring is a masked 1,3-amino alcohol and served as a convenient synthon for ring-opening reactions with a variety of nucleophiles, including phenoxides, alkoxides, and thiols.^18^

## 3. Enantioselective *aza*-Wacker cyclization reactions

Examples of enantioselective *aza*-Wacker cyclization reactions remain sparse in the literature. This is a highly challenging transformation to design for multiple factors, including the possibility of both *cis* and *trans* nucleopalladation as well as a general lack of asymmetric ligands for the Pd(ii)–Pd(0) redox manifold.^19^ The purpose of this section is to summarize what exists of the intramolecular asymmetric *aza*-Wacker reaction.

### 3.1 Enatioselective synthesis of indolines through tandem C–N and C–C bond formation (2006)

**Fig. 17:**
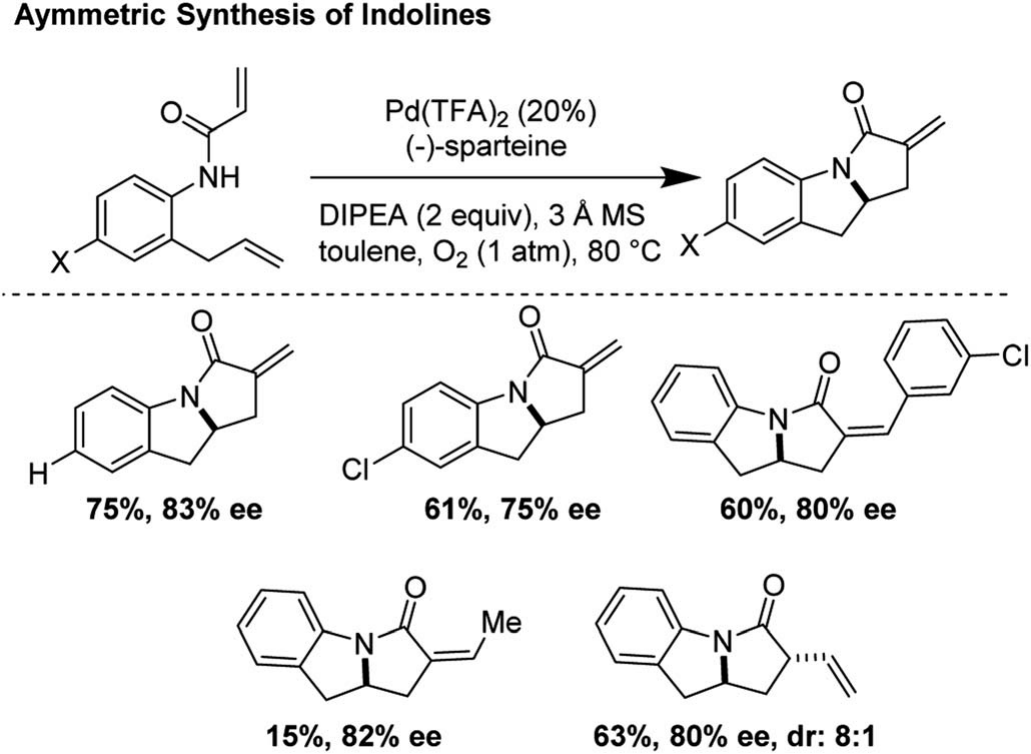
Asymmetric synthesis of indolines *via* a tandem *aza*-Wacker cyclization/carbopalladation sequence.

A pioneering example of an asymmetric *aza*-Wacker type cyclization reaction was reported by Yang and co-workers in 2006. Using the reagent combination of Pd(TFA)_2_/(−)-sparteine, tricycles were rapidly synthesized with enantiomeric excesses ranging from 75–86% (Fig. 17).^20^ In one step, a new C–N bond is forged with reasonable enantioselectivity followed by formation of a new C–C bond through a carbopalladation mechanism. The Yang laboratory has worked to significantly expand the scope of this transformation through ligand design; in subsequent reports, they demonstrated that Pd(TFA)_2_/(*S*,*S*)-diPh-pyridine-oxazoline and Pd(OAc)_2_/t-Bu-quinoline-oxazoline allowed for cascade cyclizations of a diverse array of diene substrates in good yields and high enantioselectivities.^21,22^

### 3.2 Enantioselective *aza*-Wacker cyclization reaction of tosylated aryl amines (2010)

**Fig. 18:**
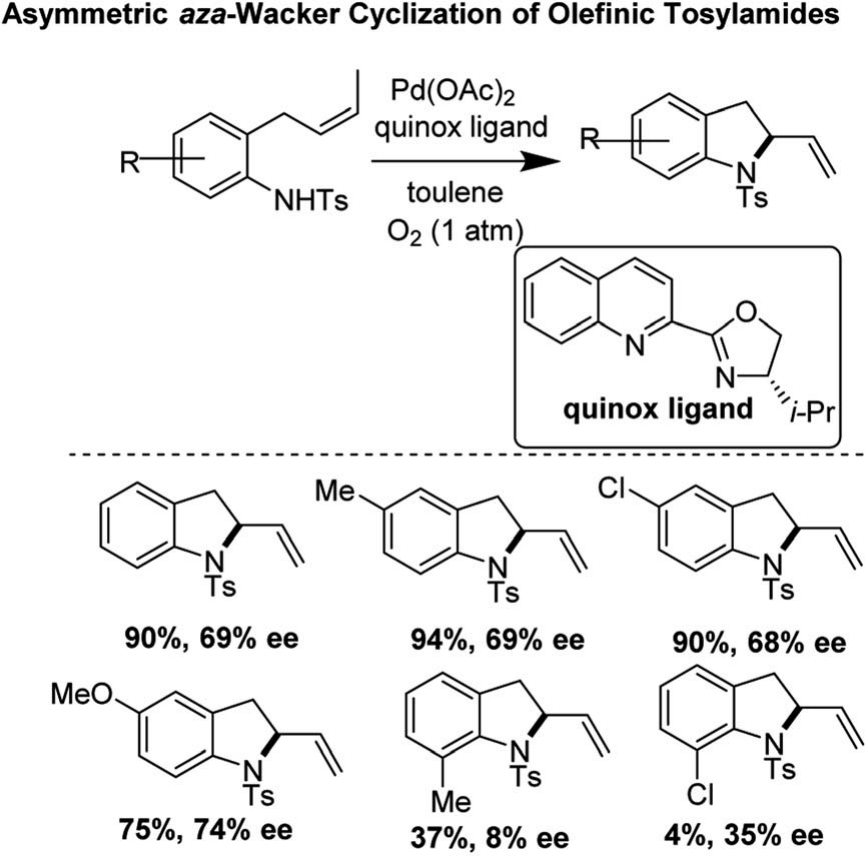
Asymmetric cyclization of olefinic tosylamides using Pd(OAc)_2_/quinox.

Zhang and co-workers reported a pioneering example of an asymmetric *aza*-Wacker cyclization reaction of olefinic tosylamides using the reagent combination of Pd(OAc)_2_/quinox ligand under 1 atm O_2_ in toluene (Fig. 18).^23^ They found that the best enantioselectivities (74% ee) were achieved with *para*-substituted substrates; enantioselectivity markedly worsened with substrates with *ortho*-substituents.

### 3.3 Enantioselective *aza*-Wacker cyclization reaction of tosylated alkyl amines (2011)

**Fig. 19:**
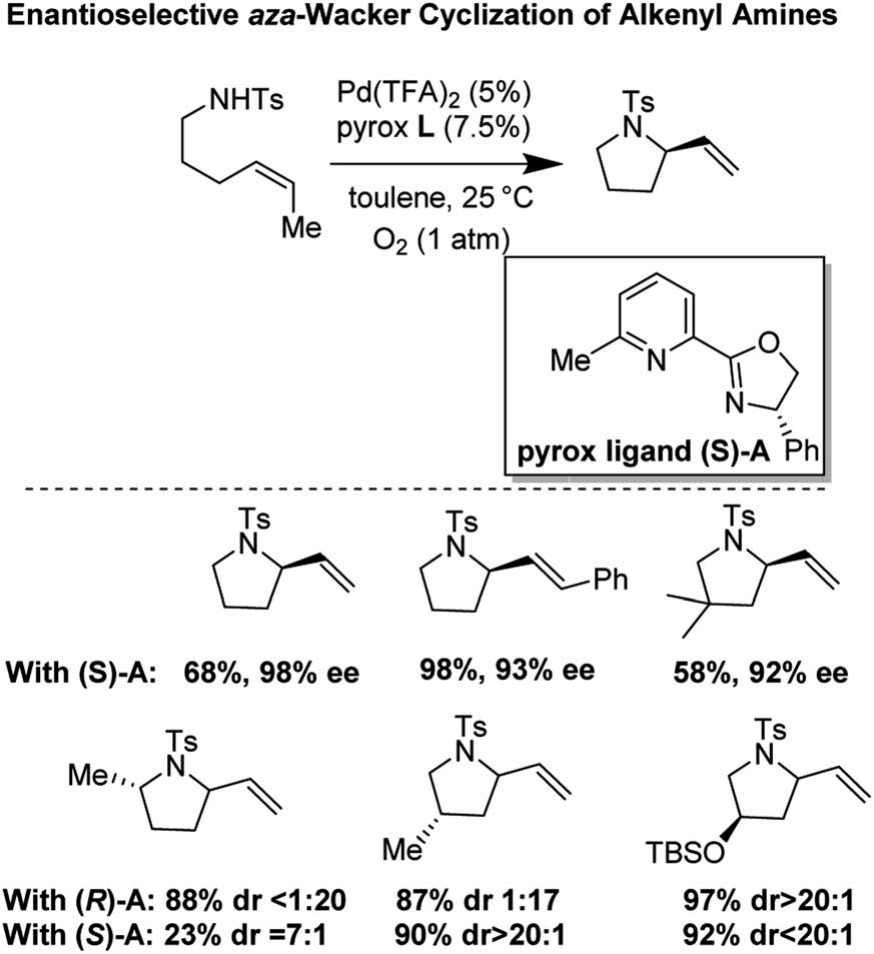
Stahl's enantioselective cyclization of alkenyl amines.

**Fig. 20:**
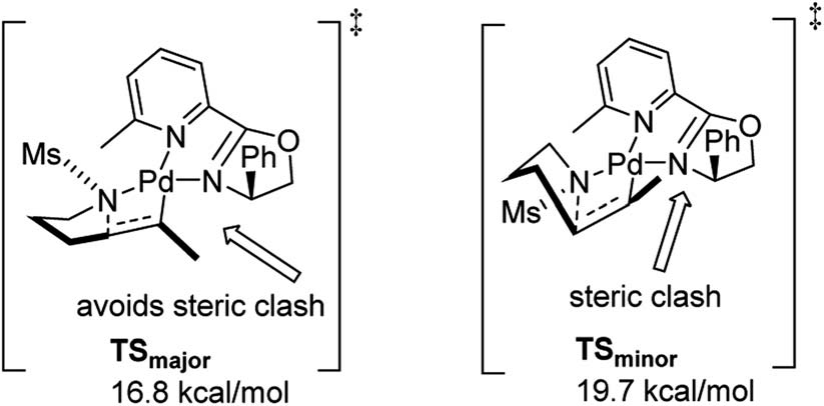
Calculated transition state for major and minor enantiomers.

In 2011, Stahl and co-workers disclosed a highly enantioselective cyclization reaction of tosylated alkyl amines using a Pd(TFA)_2_/pyrox manifold (Fig. 19).^24,25^ The reactions proceeded at room temperature and furnished cyclized alkenyl pyrrolidine products in good yields (∼60–98%) and excellent enantioselectivities (ee = 92–98%). By examining cyclization reactions with racemic pyrox ligand as well as with the (*R*) and (*S*) antipodes individually, they discovered that the nature of the ligand could override diastereomeric bias conferred by the inherent steric and electronic properties of the substrate. Extensive DFT computations revealed that the transition state leading to the major enantiomer is approximately 3 kcal mol^−1^ lower than that leading to the minor one (Fig. 20).

### 3.4 Enantioselective *aza*-Wacker cyclization for the synthesis of isoindolinones (2012)

**Fig. 21:**
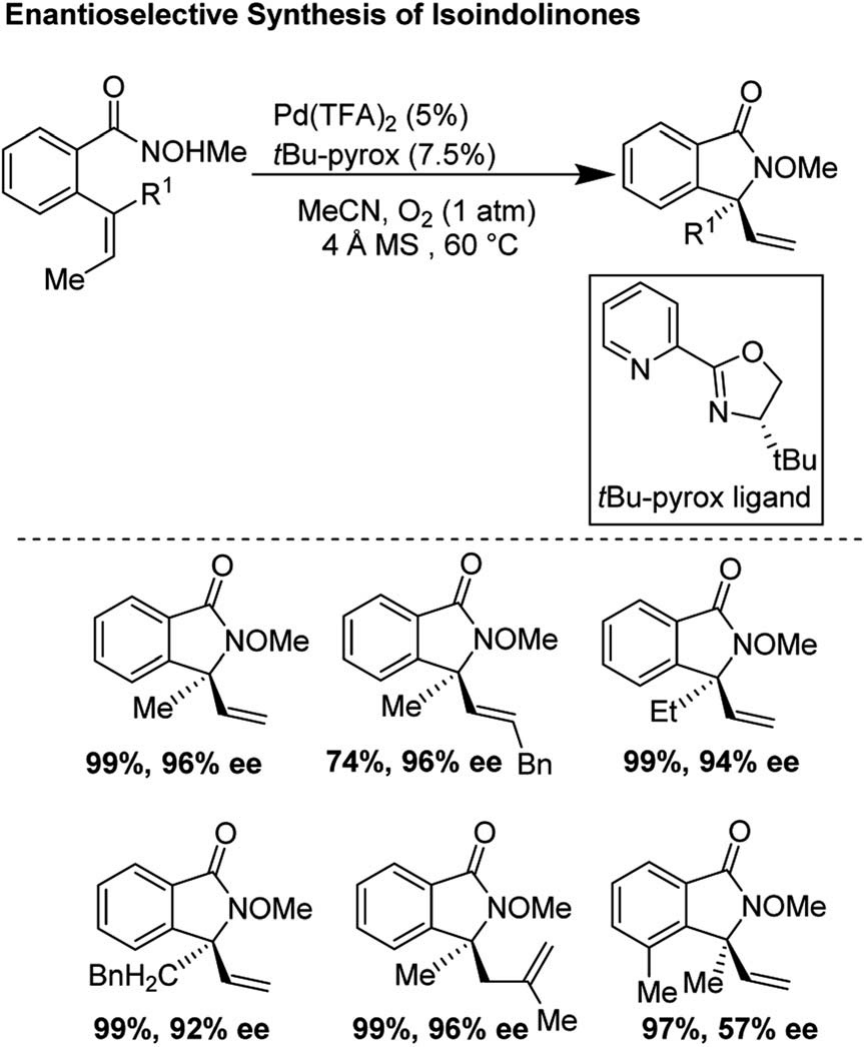
Zhang's enantioselective synthesis of isoindolinones.

**Fig. 22:**
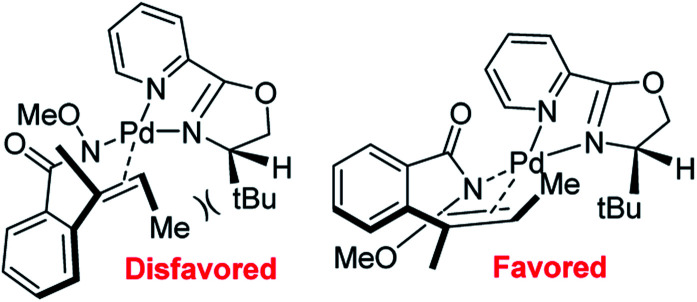
Zhang's model for asymmetric induction.

In 2012, Zhang and co-workers disclosed a highly enantioselective *aza*-Wacker cyclization which furnishes isoindolinones bearing quaternary stereocenters (Fig. 21).^26^ After testing a variety of quinox and pyrox ligands in combination with Pd(ii) salts, they discovered that the reagent manifold of Pd(TFA)_2_/*t*Bu-pyrox under 1 atm of O_2_ in CH_3_CN furnished products with excellent yields and enantioselectivities (ee up to 99%). Importantly, they found that the *N*-OMe is cleaved facilely using SmI_2_ yielding *N*-unfunctionalized amide products. They hypothesized that the N-atom of the methoxyamine auxiliary is bound to the Pd center, *cis* to the pyridine moiety of the pyrox ligand; the substrate olefin is thus bound *trans* to the pyridine moiety (Fig. 22). If this olefin has *Z* geometry, the terminal methyl group is oriented up and away from the pyrox *tert*-butyl group, allowing for strong asymmetric induction. Finally, *syn*-amino-palladation and β-hydrogen elimination furnishes product.

### 3.5 Enantioselective *aza*-Wacker cyclization for the synthesis of 6-membered heterocycles (2018)

**Fig. 23:**
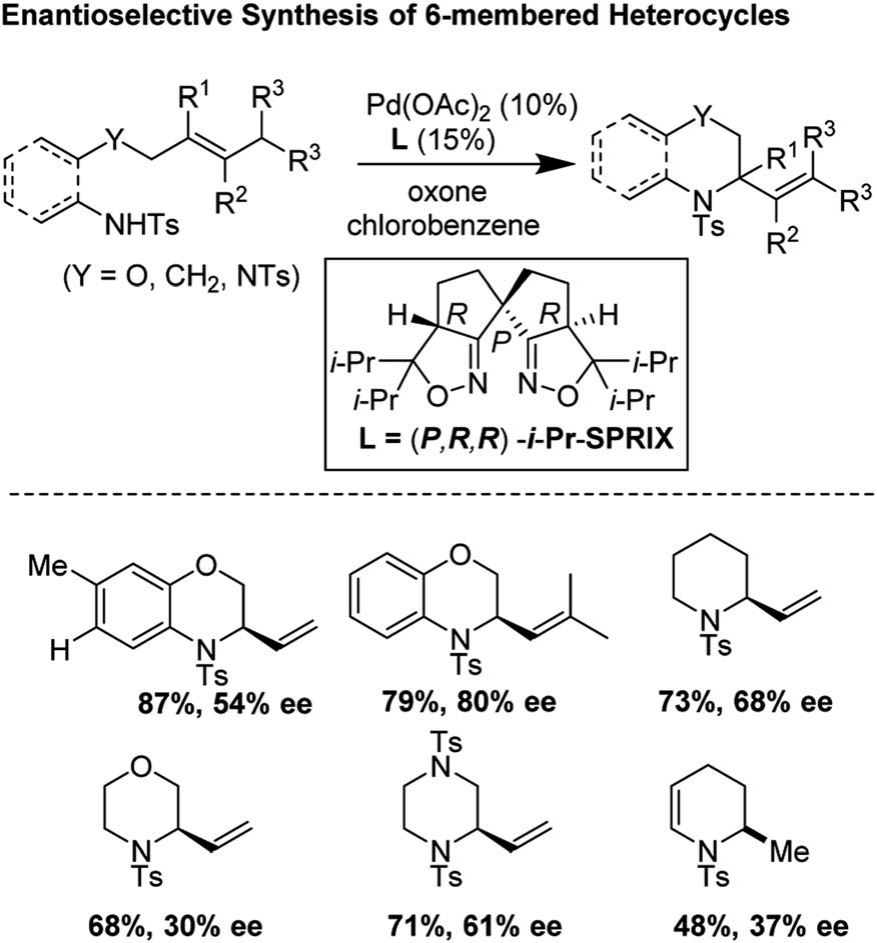
Asymmetric synthesis of diverse six-membered heterocycles using Pd(OAc)_2_/iPr-SPRIX.

**Fig. 24:**
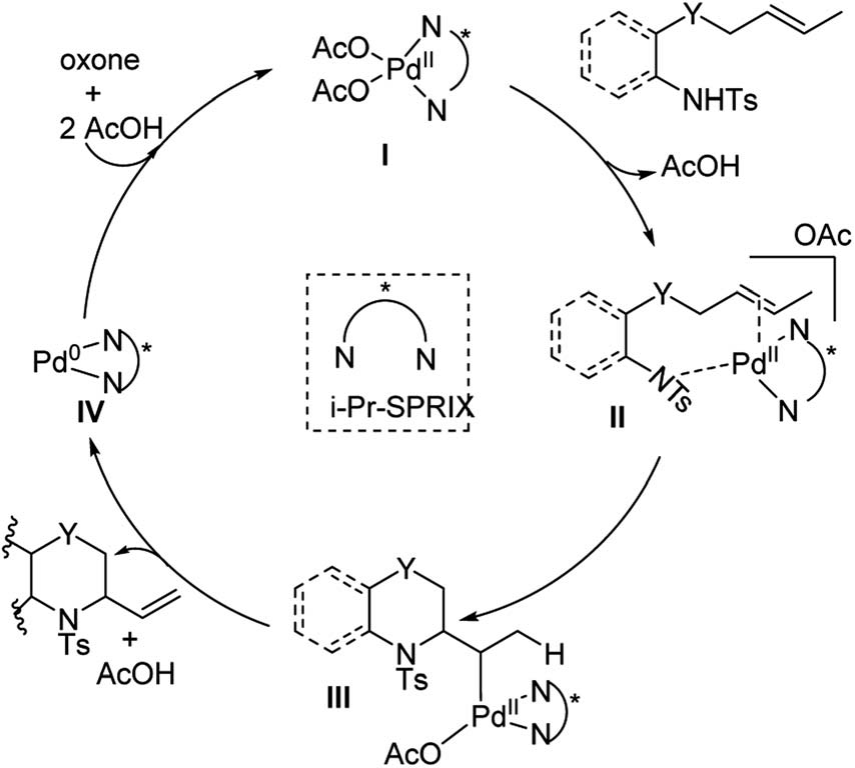
Proposed catalytic cycle.

In 2018, Sen, Takenaka, and Sasai disclosed an enantioselective synthesis of 6-membered heterocycles using an *aza*-Wacker cyclization reaction, patterned on Stahl's previous racemic disclosure in 2012 (Fig. 23).^27^ For this reaction, they used catalytic Pd(OAc)_2_ in conjunction with (*P*,*R*,*R*)-iPr-SPRIX ligand and oxone as the terminal oxidant; they had previously used the combination of Pd(ii) and SPRIX ligands for Yang-type cascade cyclizations, but the enantioselectivities of these reactions did not cross 61%.^28^ They hypothesized that the low σ-donor ability of the SPRIX ligand allows for preservation of the Lewis acidity of the Pd(OAc)_2_ and efficient alkene activation. Survey of a variety of alkenyl substrates revealed that the presence of a bulky substituent at the olefin terminus was critical for efficient enantio-induction. Sasai and co-workers conjectured that avoidance of unfavorable steric clashing between bulky groups on the olefin and the isopropyl groups of the SPRIX ligand allowed for olefin facial discrimination during the critical *syn*-amino-palladation event (Fig. 24). Under optimal conditions, they were able to achieve yields as high as 87% with enantioselective excesses as large as 80%.

### 3.6 Enantioselective synthesis of 6,5-bicyclic *aza*-heterocycles (2014)

**Fig. 25:**
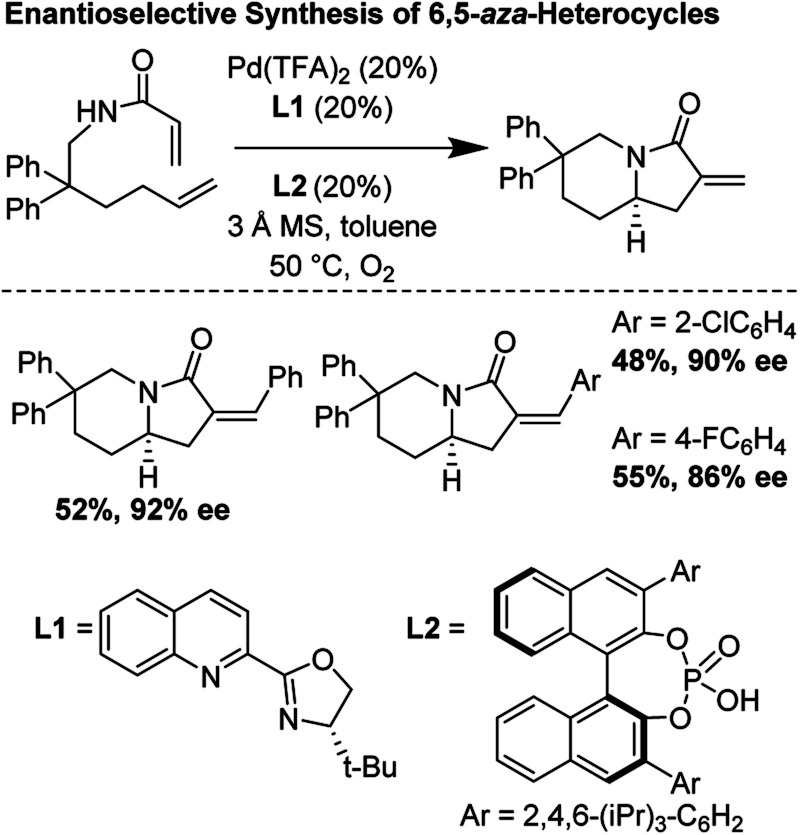
Gong's enantioselective synthesis of 6,5-heterocycles through tandem cyclization reactions.

Building upon foundational work by the Yang laboratory (*vide supra*), in 2014, Gong and co-workers disclosed a highly enantioselective oxidative tandem cyclization reaction which furnished 6,5-bicyclic *aza*-heterocycles in good yields and enantioselectivities up to 92%.^29^ They found that the unusual combination of Pd(TFA)_2_, *t*Bu-quinolineoxazoline, and a chiral phosphoric acid additive were essential for reaction optimization (Fig. 25).

### 3.7 Enantioselective synthesis of indolines (2019)

**Fig. 26:**
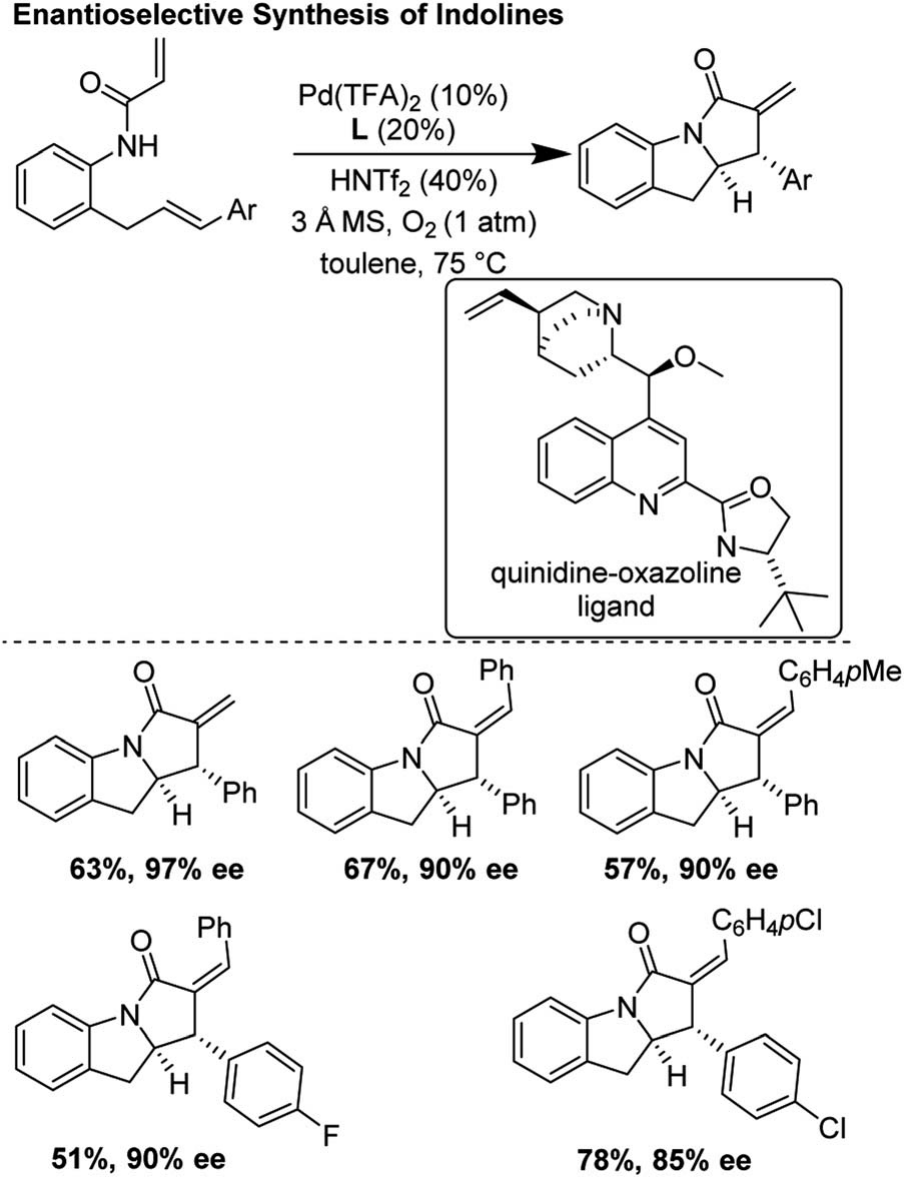
He's improved asymmetric synthesis of indolines.

He and co-workers recently improved upon the tandem *aza*-Wacker carbopalladation sequence first reported by Yang and co-workers in 2006.^30^ In this report, they developed a new class of chiral ligand, the quinidine oxazoline, and applied it in an asymmetric tandem *aza*-Wacker carbopalladation cascade (Fig. 26). Using this exciting new ligand framework, they were able obtain enantioselective excesses up to 97%, with diastereomeric ratios generally greater than 20 : 1. For select substrates, the reaction scaled well, with little loss in yield and enantioselectivity.

## 4. *aza*-Wacker cyclization reactions applied to natural products synthesis

There are very few examples of *aza*-Wacker cyclization reactions applied as key steps in the synthesis of complex natural products. This is in sharp contrast to other alkene and alkane oxidation reactions, especially C–H amination.^31^ We find this to be a missed opportunity because intramolecular *aza*-Wacker reactions offer great precision in the site-selective functionalization of alkene moieties. Not only is a new C–N bond formed but also the alkenyl moiety is transposed into a new location, essentially serving as a blank slate for a variety of subsequent mono- and di-functionalization reactions.

In this portion, we summarize existing examples of *aza*-Wacker cyclization reactions utilized in the pursuit of complex natural products. Most of these examples are *aza*-Wacker reactions employing native amines or amides; the lone exception is Stahl's elegant assembly of (−)-acosamine, which highlights the use of a tethered *aza*-Wacker reaction. The wider adoption of *aza*-Wacker reactions by the synthetic community would represent an important shift in the logic of nitrogen insertion during complex molecule assembly.

### 4.1 Total synthesis of (±)-bukittinggine

Bukittinggine is *Daphniphyllum* alkaloid with potent anti-inflammatory activity that was first isolated from the leaves and branches of *Sapium baccatum* collected near the town of Bukittinggi, West Sumatra, Indonesia.^32^ Of this large family of alkaloids, bukittinggine is one of the only members with a heptacyclic framework, providing a challenging target for synthetic chemistry efforts.

**Fig. 27:**
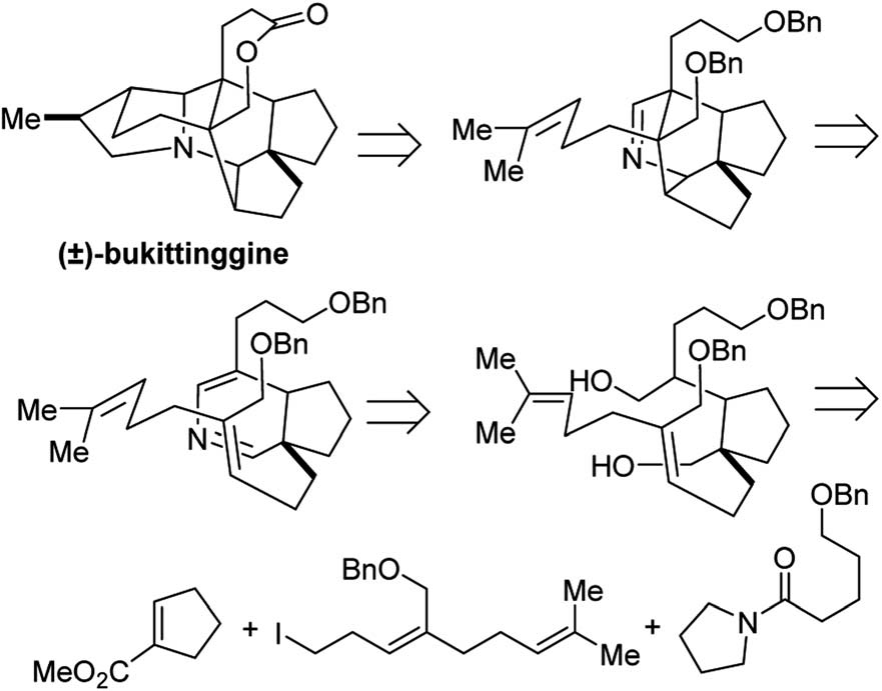
Bukittinggine is a complex heptacyclic alkaloid.

**Fig. 28:**
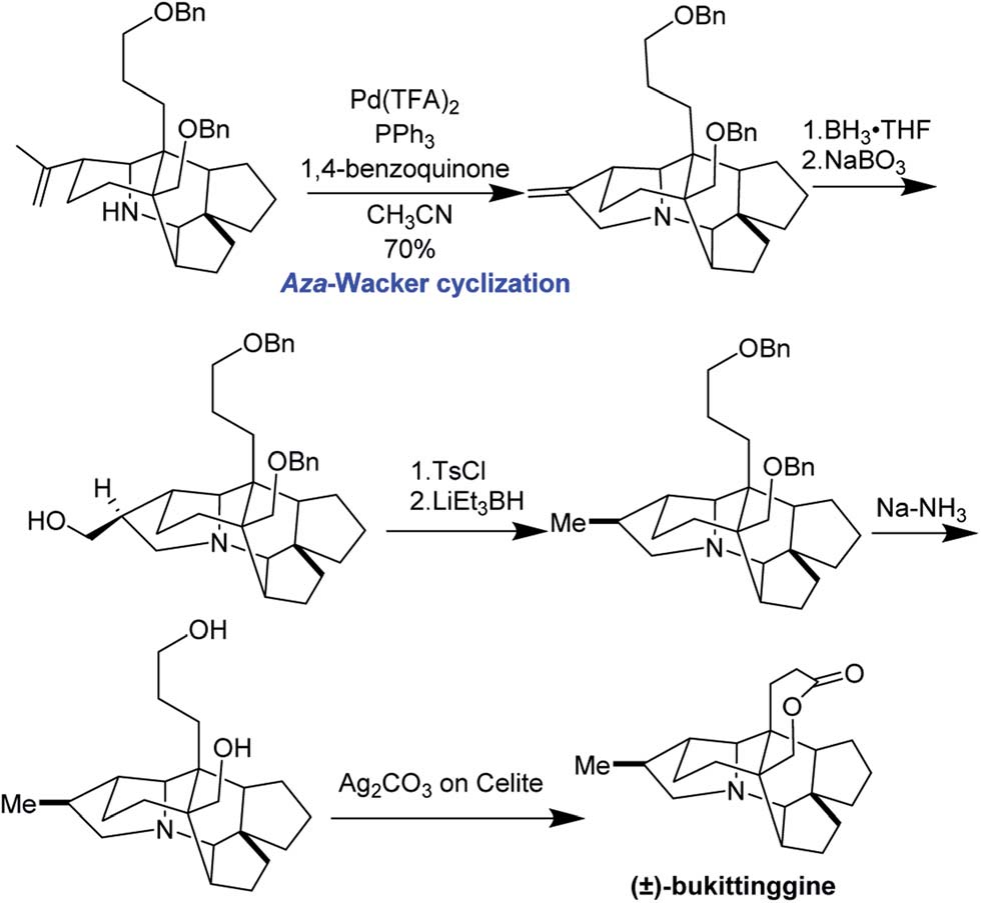
The *aza*-Wacker cyclization is a key step in Heathcock's synthesis.

Clayton H. Heathcock and co-workers achieved the first total synthesis (±)-bukittinggine. Highlights of their synthesis include a marvelous intramolecular Diels–Alder reaction followed by an acid-mediated cationic cyclization to furnish the secodaphnane nucleus (Fig. 27). The pyrrolidine ring was constructed using an *aza*-Wacker oxidative cyclization reaction, one of the first applications of this reaction in natural product synthesis (Fig. 28).

During significant optimization of this reaction, the authors observed a marked improvement when using palladium(ii) trifluoroacetate relative to palladium(ii) acetate. Further, they found that the quality of benzoquinone was critical and that the reaction proceeded best when it was recrystallized prior to use. Following the *aza*-Wacker cyclization reaction, the final stereocenter was set using a diasteroselective hydroboration–oxidation reaction followed by removal of the resulting alcohol. The final lactone ring was formed through oxidation with Ag_2_CO_3_ adsorbed onto Celite.

### 4.2 Enantioselective syntheses of (−)-melinonine-E and (+)-strychnoxanthine

**Fig. 29:**
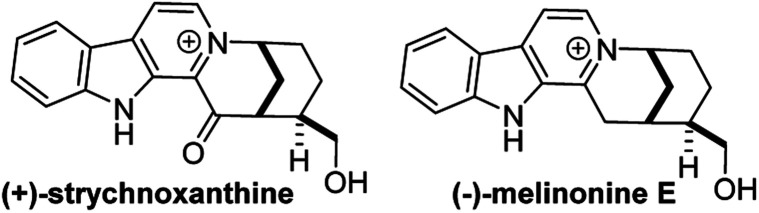
β-Carbolinium alkaloids with a fused 2-azabicyclo[3.3.1]nonane skeleton.

β-Carbolinium alkaloids are a large family of structurally interesting, biologically active natural products (Fig. 29). Melinonine-E was first isolated in 1957 from the bark of *Strychnos melinoniana* Baillon (Loganiaceae), a plant used in African folk medicine for malarial therapy.^33^ A related natural product, strychnoxanthine, was isolated by Angenot and co-workers from *Strychnos gossweileri* Exell (Loganiaceae);^34^ importantly, strychnoxanthine was found to inhibit *Plasmodium falciparum* (IC_50_ = 8.4 μm).^35^ Both melinonine-E and strychnoxanthine are pentacycles with a β-carbolinium motif fused to a 2-*aza*bicyclo[3.3.1]nonane skeleton.

**Fig. 30:**
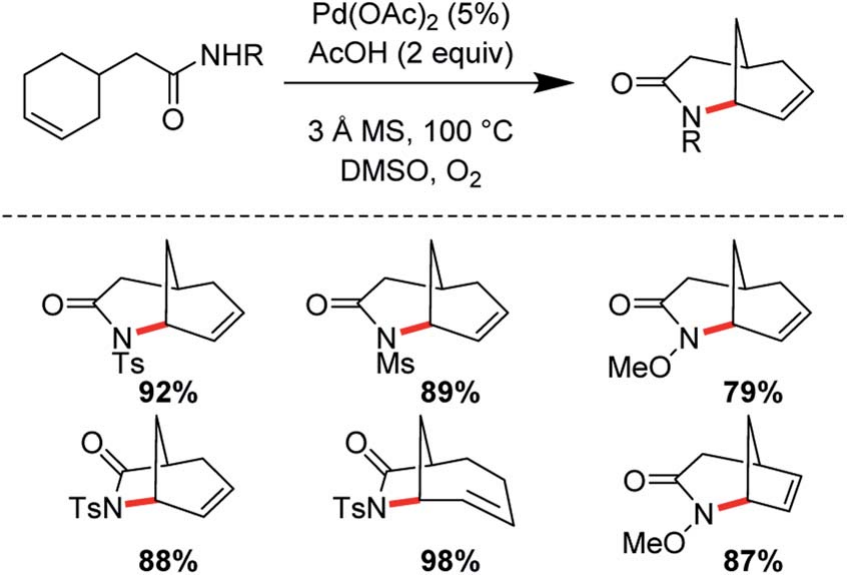
Hong's *aza*-Wacker cyclization constructs bicyclic rings.

**Fig. 31:**
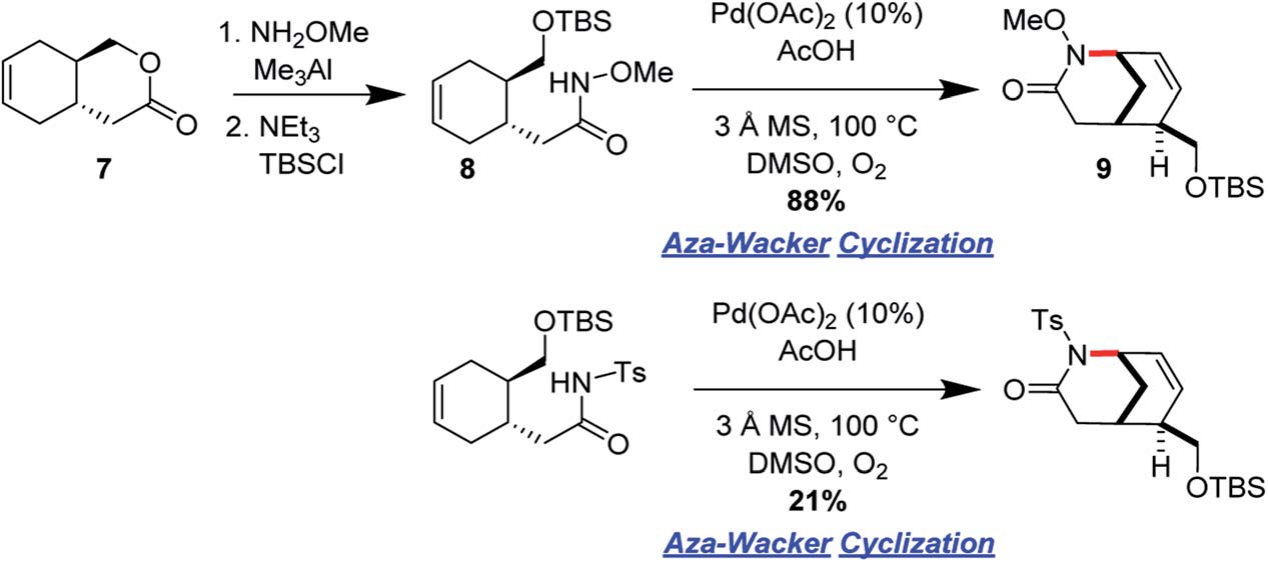
Hong's *aza*-Wacker cyclization reaction is exquisitely sensitive to steric and electronic factors.

**Fig. 32:**
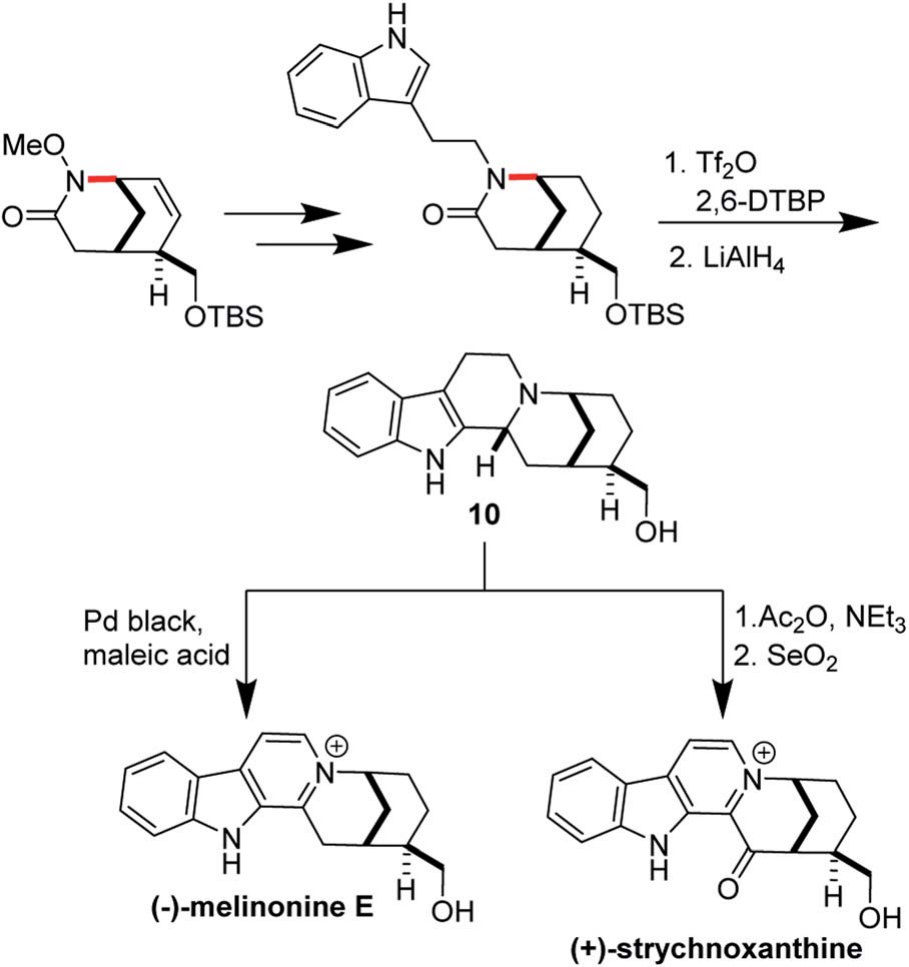
Endgame of the melinonine and strychnoxanthine synthesis.

Ran Hong and co-workers furnished the first asymmetric syntheses of (−)-melinonine-E and (+)-strychnoxanthine; a key step in both syntheses was an elegant *aza*-Wacker cyclization to construct the bicycle (Fig. 30).^36,37^ Cyclization precursor **8** was constructed from chiral lactone **7** in 2 steps; this lactone had previously been synthesized by Hong and co-workers as a key intermediate in the construction of other alkaloid natural products.^38^ Interestingly, the *N*-substituent had a marked effect on the success of the *aza*-Wacker cyclization. When the OMe group was replaced by a tosyl substituent, the yield dropped significantly (Fig. 31). The resulting bicycle was expeditiously converted into (−)-melinonine E in a series of five steps, with a powerful Tf_2_O/2,6-DTBP cyclodehydration reaction as a highlight (Fig. 32). Intermediate **10** was converted into (+)-strychnoxanthine after acylation and SeO_2_ oxidation (Fig. 32).

### 4.3 Enantioselective synthesis of (−)-arcutinine

(−)-Arcutinine is a C_20_ diterpenoid alkaloid molecule, a member of a much larger family of C_18_, C_19_, and C_20_ natural products isolated from the *Acontium*, *Delphinium*, and *Spiraea* genera of plants.^39^ Arcutine-type alkaloids are characterized by a challenging, functional group adorned hexacyclic framework with three all-carbon quaternary centers. (−)-Arcutinine was first isolated by Saidkhodzhaeva and co-workers from *Aconitum arcuatum*.^40^

**Fig. 33:**
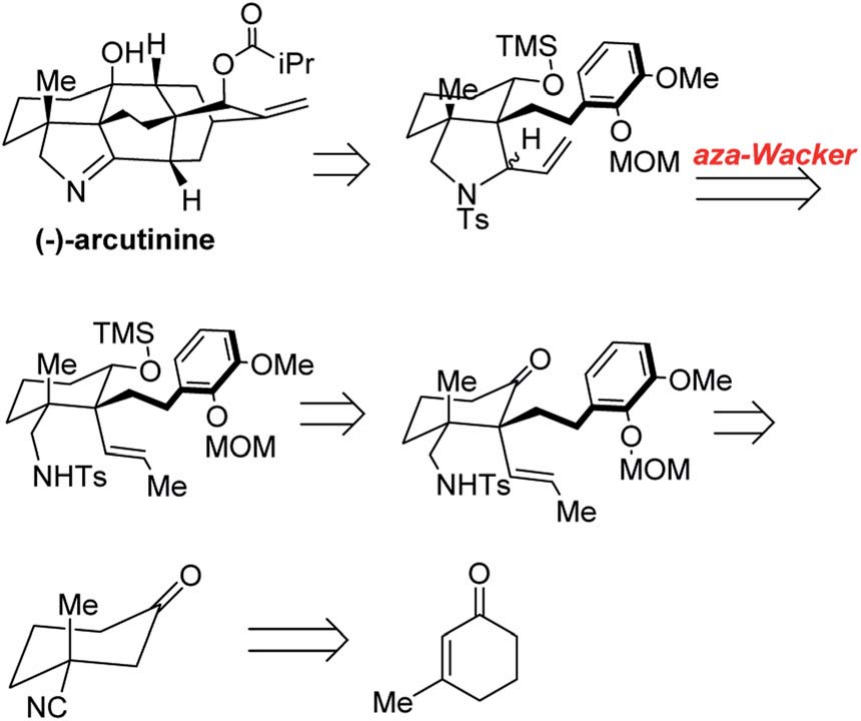
Yong Qin's retrosynthesis of (−)-arcutinine.

**Fig. 34:**
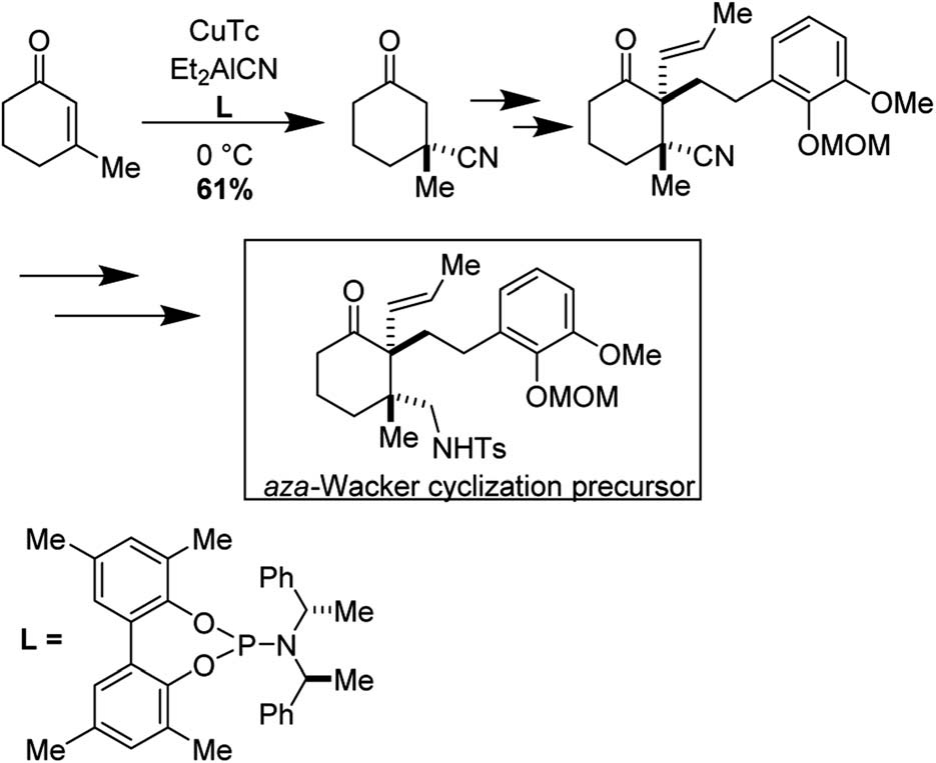
Opening sequence of reactions sets up the *aza*-Wacker cyclization.

Yong Qin and co-workers realized the first asymmetric synthesis of (−)-arcutinine using the Stahl *aza*-Wacker cyclization reaction^41^ as a key step (Fig. 33). The cyclization precursor was assembled in several steps from 1,3-cyclohexanedione; highlights of this sequence include an asymmetric conjugate addition of TMS cyanide, a diastereoselective palladium-catalyzed decarboxylative allylation, and a palladium-catalyzed olefin isomerization (Fig. 34).

**Fig. 35:**
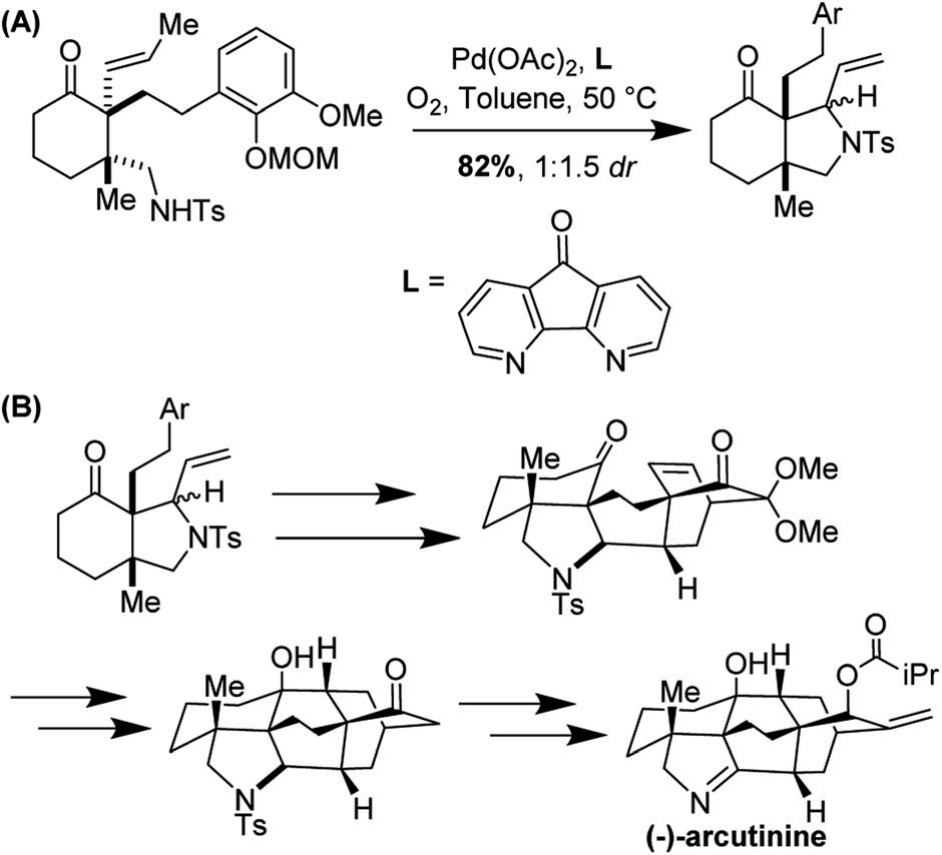
(A) *aza*-Wacker cyclization using Stahl conditions (B) elaboration into (−)-arcutinine.

The *aza*-Wacker cyclization proceeded with a reasonable diastereoselectivity of 2.5 : 1 in a total yield of 76%. The resulting product was elaborated into (−)-arcutinine in a dramatic sequence of reactions, of which an oxidative dearomatization/intramolecular Diels–Alder cascade and a ketyl–olefin cyclization are highlights (Fig. 35).

### 4.4 Enantioselective synthesis of (−)-acosamine

**Fig. 36:**
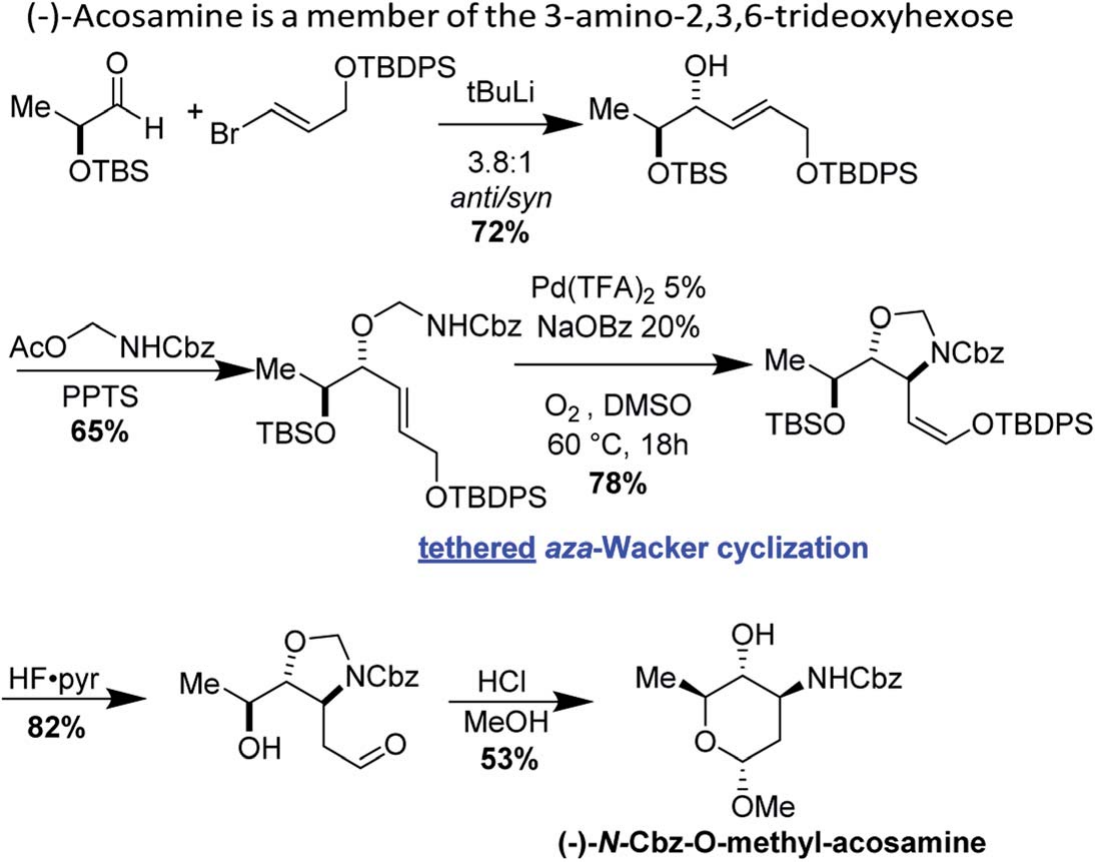
Stahl's synthesis of protected (−)-acosamine.

(−)-Acosamine is a member of the 3-amino-2,3,6-trideoxyhexose sugars, important elements of antibacterial and antitumor antibiotics.^42^ Shannon Stahl and co-workers have reported an elegant synthesis of (−)-acosamine, applying their hemiaminal tethered *aza*-Wacker cyclization reaction (Fig. 36).^13^ This synthesis is also notable as it remains the lone example of a tethered *aza*-Wacker cyclization applied to the assembly of a natural product.

Stahl's synthesis commences from TBS-protected (−)-lactaldehyde and furnishes the hemiaminal precursor for the key *aza*-Wacker cyclization reaction in two steps. The hemiaminal tethered *aza*-Wacker cyclization reaction proceeds in good yield and excellent diastereoselectivity to furnish a cyclic 5-membered *N*,*O*-acetal. Silyl deprotection followed by acid mediated ring closure furnishes (−)-*N*-Cbz-*O*-methyl-acosamine, a convenient surrogate of (−)-acosamine.

### 4.5 Enantioselective synthesis of (−)-mesembrane and (+)-crinane

**Fig. 37:**
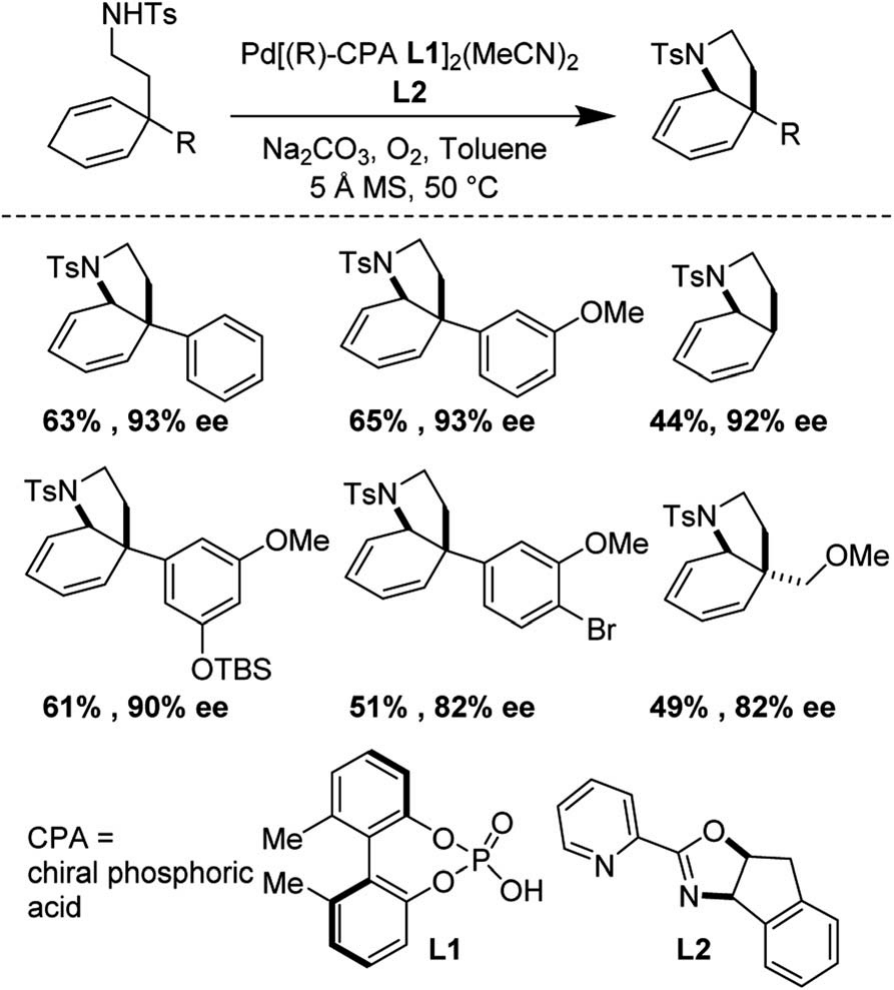
Zhu's desymmetrizing *aza*-Wacker cyclization.

(−)-Mesembrane and (+)-crinane are alkaloid natural products which are present in the *Amaryllidaceae* plant family. Their *cis*-3a-aryloctahydroindole framework, decorated with a variety of quaternary and tertiary sterocenters, has attracted much attention from the synthetic community.^43,44^ Jieping Zhu and co-workers have recently realized enantioselective syntheses of (−)-mesembrane and (+)-crinane by applying an elegant catalytic, enantioselective, desymmetrizing *aza*-Wacker cyclization reaction (Fig. 37).^45^ It should be noted that the authors discovered that for the best enantioselectivity, *two* chiral ligands were necessary, one pre-complexed to the palladium catalyst and the other added into the reaction mixture.

**Fig. 38:**
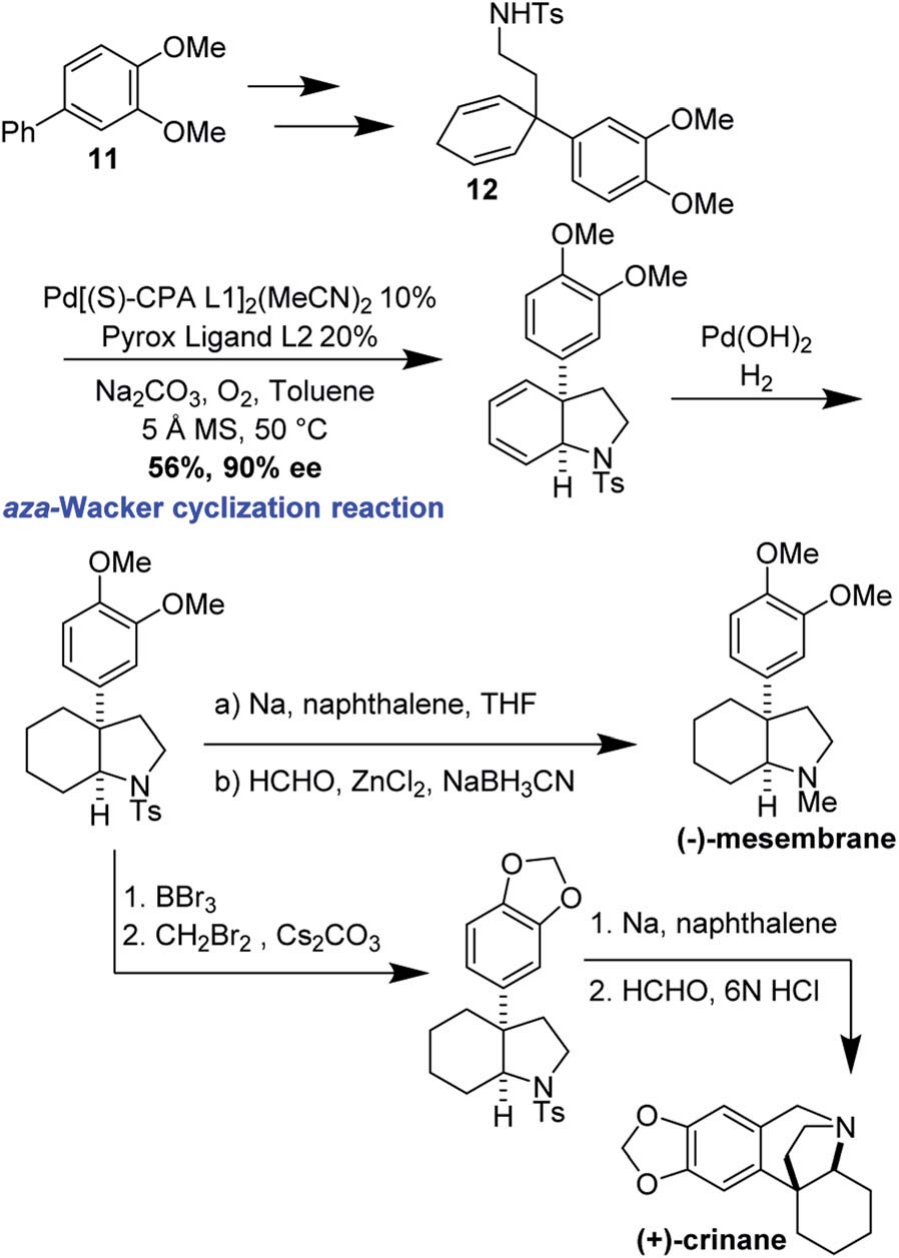
Zhu's synthesis of (−)-mesembrane and (+)-crinane.

Cyclization precursor **12** was synthesized in three steps from biaryl **11**. Zhu's desymmetrizing *aza*-Wacker cyclization proceeded in reasonable yield and enantioselectivity. From this common intermediate, the synthesis of (−)-mesembrane proceeded by removal of the tosyl group and subsequent reductive amination. BBr_3_ di-demethylation, methylenation of the resulting catechol, tosyl removal, and final Pictet–Spengler cyclization afforded (+)-crinane (Fig. 38).

### 4.6 Enantioselective synthesis of (−)-mitomycin K

**Fig. 39:**
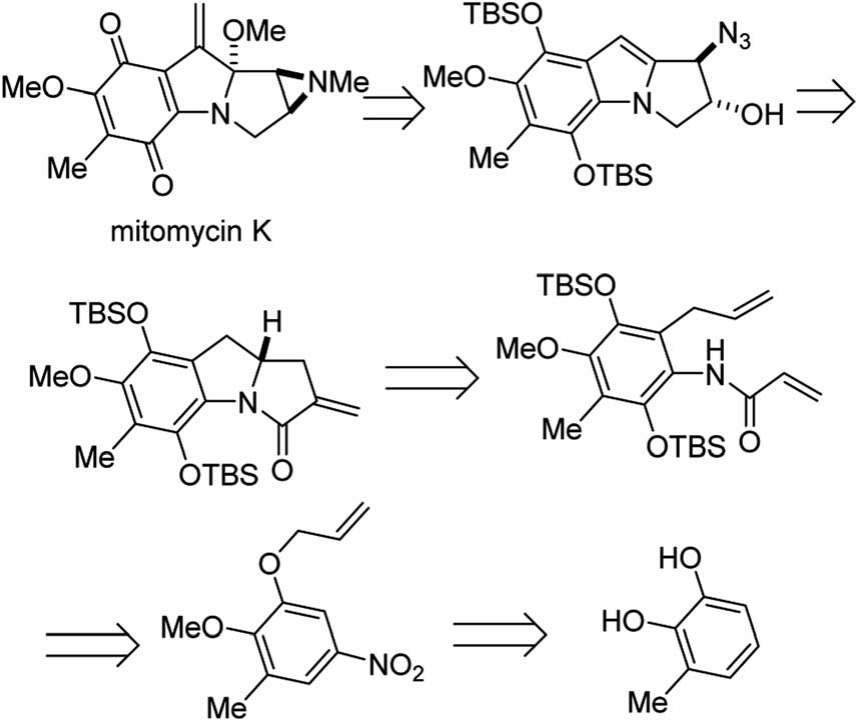
Mitomycin K is a potent antitumor antibiotic Yang's retrosynthesis is shown here.

Mitomycins are a family of aziridine-containing antitumor and antibacterial natural products isolated from culture broths of *Streptomyces caespitosus* and *Streptomyces lavendulae*.^46^ One member of this family, mitomycin C, is FDA approved for the treatment of urothelial carcinoma^47^ as well as to prevent postoperative scarring following trabeculectomy to relieve intraocular pressure in glaucoma.^48^ Dan Yang and co-workers recently succeeded in the first enantioselective synthesis of another member of this remarkable family, mitomycin K, using an elegant *aza*-Wacker type amination carbopalladation cascade.^49^ In one step, this reaction forms a new C–N bond with high enantioselectivity followed by a new C–C bond with high diastereoselectivity (Fig. 39).

**Fig. 40:**
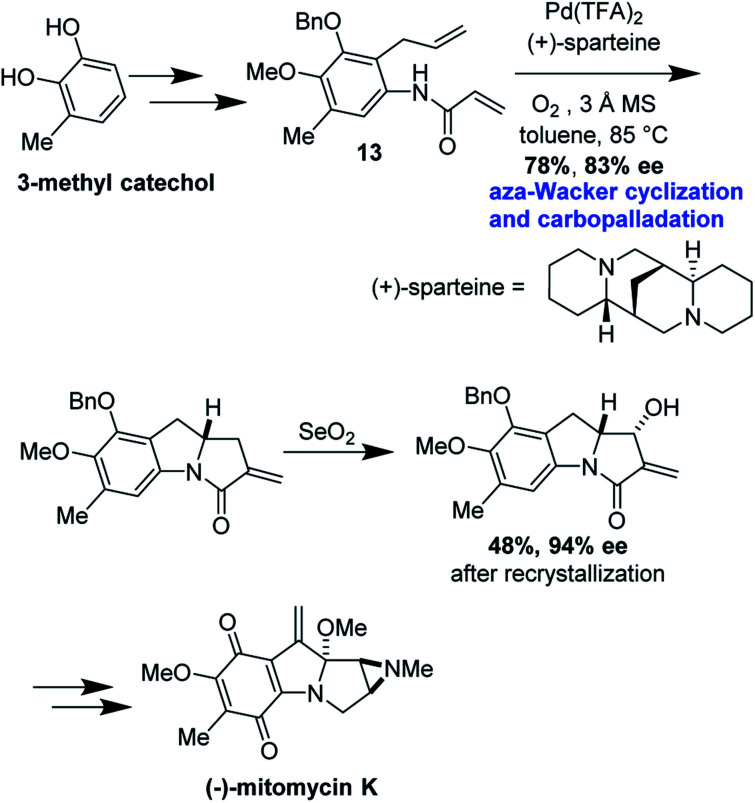
Mitomycin K forward synthetic route.

Cyclization precursor **13** was assembled in several steps from commercially available 3-methylcatechol (Fig. 40). After significant optimization, the authors found that the combination of Pd(TFA)_2_/(+)-sparteine afforded the desired tricycle in 78% yield, 83% ee; this was increased to 94% after recrystallization in the next step. This tricycle served as a key intermediate for elaboration into (−)-mitomycin K.

## 5. Conclusion and outlook

Several aspects of the intramolecular *aza*-Wacker alkene functionalization reaction are highlighted herein. We first drew the distinction between traditional *aza*-Wacker cyclizations and tethered *aza*-Wacker reactions and summarized existing examples of the latter. We next focused on the development of asymmetric *aza*-Wacker cyclizations, an emerging and exciting subfield. Finally, we summarized applications of *aza*-Wacker reactions in the synthesis of natural products. Where applicable, mechanistic details of each reaction were discussed. While *aza*-Wacker cyclizations have been elegantly developed by efforts world-wide, much work remains before they join the pantheon of the most prized organic transformations, such as asymmetric hydrogenation, cross-coupling, and olefin metathesis. In particular, there are few examples of tethered *aza*-Wacker cyclization methods, and their applications to total synthesis are also limited. Collectively, we hope that this review will serve to inspire the development of new *aza*-Wacker reactions for creative applications in complex molecule assembly.

## Conflicts of interest

There are no conflicts to declare.
